# Bacterial persisters: molecular mechanisms and therapeutic development

**DOI:** 10.1038/s41392-024-01866-5

**Published:** 2024-07-17

**Authors:** Hongxia Niu, Jiaying Gu, Ying Zhang

**Affiliations:** 1 School of Basic Medical Science and Key Laboratory of Blood-stasis-toxin Syndrome of Zhejiang Province, Zhejiang Chinese Medical University, Hangzhou, 310053 Zhejiang China; 2 State Key Laboratory for the Diagnosis and Treatment of Infectious Diseases, The First Affiliated Hospital of Zhejiang University School of Medicine, Hangzhou, 310003 Zhejiang China; 3 Jinan Microecological Biomedicine Shandong Laboratory, Jinan, 250022 Shandong China

**Keywords:** Microbiology, Target identification

## Abstract

Persisters refer to genetically drug susceptible quiescent (non-growing or slow growing) bacteria that survive in stress environments such as antibiotic exposure, acidic and starvation conditions. These cells can regrow after stress removal and remain susceptible to the same stress. Persisters are underlying the problems of treating chronic and persistent infections and relapse infections after treatment, drug resistance development, and biofilm infections, and pose significant challenges for effective treatments. Understanding the characteristics and the exact mechanisms of persister formation, especially the key molecules that affect the formation and survival of the persisters is critical to more effective treatment of chronic and persistent infections. Currently, genes related to persister formation and survival are being discovered and confirmed, but the mechanisms by which bacteria form persisters are very complex, and there are still many unanswered questions. This article comprehensively summarizes the historical background of bacterial persisters, details their complex characteristics and their relationship with antibiotic tolerant and resistant bacteria, systematically elucidates the interplay between various bacterial biological processes and the formation of persister cells, as well as consolidates the diverse anti-persister compounds and treatments. We hope to provide theoretical background for in-depth research on mechanisms of persisters and suggest new ideas for choosing strategies for more effective treatment of persistent infections.

## Introduction

Bacterial infections have long been a scourge for humanity. In the past century, the discovery and widespread use of antibiotics have somewhat improved the treatment of these infections.^1–3^ However, in recent decades, the emergence and increasing antibiotic resistance has heightened concerns regarding infectious disease.^4–8^ The constantly emerging phenomenon of “superbugs” in clinical setting is a sign that we are entering an era where traditional infection treatments are becoming increasingly ineffective.^9–11^ In addition to antibiotic resistance, persistent infections pose another major challenge and problem in managing bacterial infections.^12–15^ Individuals with persistent infections endure the continuous presence or recurring episodes of bacterial infections, often with poor response to antibiotic therapy.^16^ Clinical examples of such infections include tuberculosis,^17^ and typhoid fever,^18^ Lyme disease,^19^ recurrent urinary tract infections^20^ and others. The presence of bacterial persisters during infections is the main culprit behind relapse and treatment failure of persistent infections.^14,21–23^

Persisters are non-growing or slow growing bacteria that can continue to survive under “stress” conditions such as antibiotics, reactive oxygen, acid pH, or starvation. After “stress” removal, persisters can continue to grow and remain sensitive to the same “stress”.^24^ Due to their tolerance ability to antibiotics and subsequent failure in antibiotic treatments, persisters are of high clinical importance for a range of microbial pathogens. In the past three decades, researchers have made significant progress in our understanding of molecular basis of bacterial persistence,^25,26^ and established specific methods for isolation and analysis of persisters. Therefore, it is necessary to make a comprehensive and systematic review on bacterial persisters, so as to point out the direction for further research and accelerate the path for the control of persistent infections.

Although there have been review articles on the research progress of bacterial persister in the past decade, they either have a long-time span, and do not cover recent research advancements,^22,24^ or they are not comprehensive enough to provide readers with a complete understanding of all aspects of persisters.^14,27,28^ For instance, Fisher et al. had summarized the correlation between persistent bacteria and clinical diseases, with focuses on the mechanisms of bacterial persistence in toxin-antitoxin modules, stringent response, bacterial communication, drug efflux, and others.^14,27,28^ However, the mechanisms discussed do not include other mechanisms of persistence such as trans-translation and protein degradation systems, metabolism of purine and amino acid, epigenetic modifications, RNA degradation, and small non-coding RNA. Additionally, the review does not cover research advancements in anti-persister drugs. Therefore, we believe that systematic review on the discovery history, characteristics, detection methods, mechanisms of persisters, and persister drugs for more effective treatment of persistent infections is necessary and important. This review will provide an update on the mechanisms and treatment of persisters, as well as put forward the potential targets for developing new drugs against persisters and challenging problems facing persister research.

Our research group has dedicated many years to investigating the mechanisms of bacterial persistence and screening drugs that target persister cells. We have proposed a “Yin-Yang” model of bacterial persistence and treatment strategy,^24^ elucidated many novel mechanisms of persistence in diverse pathogens such as *Mycobacterium tuberculosis* (*M. tuberculosis*),^29–35^ *Escherichia coli* (*E. coli*),^36–42^ *Staphylococcus aureus* (*S. aureus*),^43–46^ and *Borrelia burgdorferi* (*B. burgdorferi*),^47,48^ and identified several drugs or drug combinations that are more effective against persisters and persistent infections than the current standard treatments.^49–60^ Drawing upon our research findings and existing literature reports, we conducted this comprehensive review of the various aspects of bacterial persisters mentioned above, aiming to provide important references for future research.

## Historical overview

**Fig. 1:**
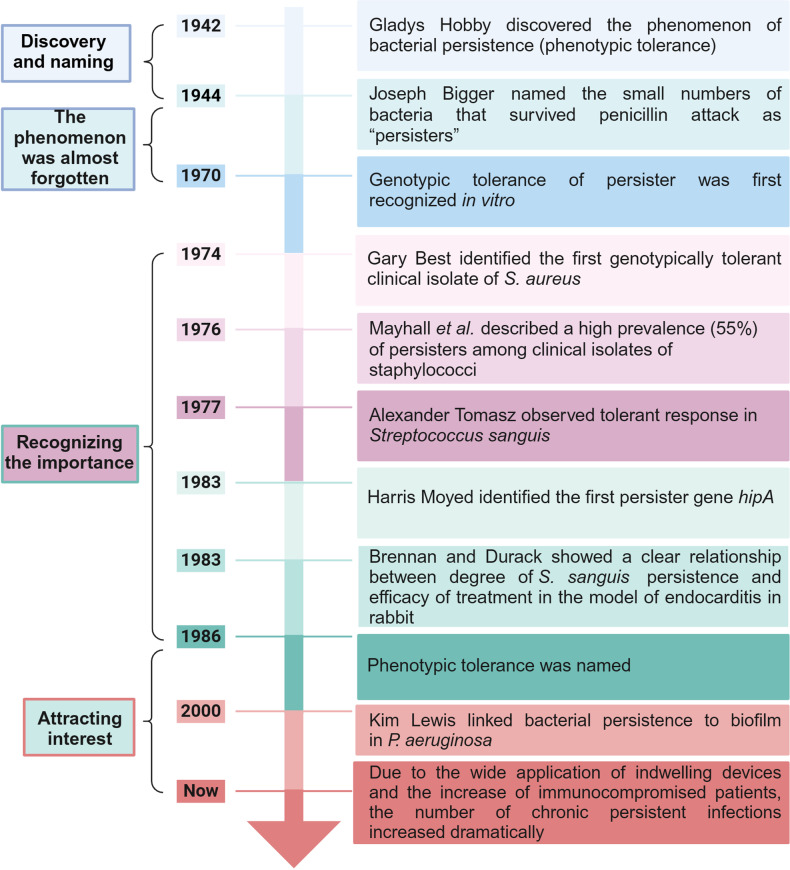
Milestone events in persister research. Since the initial discovery of bacterial persistence phenomenon in 1942, significant findings have progressively unveiled the clinical implications of bacterial persisters in human diseases. Created with BioRender.com

Persisters were first identified more than 80 years ago and are closely associated with chronic persistent infections (Fig. 1). In 1942, Gladys Hobby discovered the phenomenon of bacterial persistence when she experimented with the newly developed antibiotic penicillin and found that the drug killed 99% bacteria (pneuniococci, heniolytic streptococci and staphylococci), with 1% organisms not killed.^61^ In 1944, Joseph Bigger, who had studied the above bacterial persistence in more detail with staphylococci, named the small numbers of non-growing dormant bacteria that survived penicillin attack as “persisters”, and suggested a scheme of treatment in which penicillin is alternately administered and withheld for the treatment of bacteria in the persister phase.^62^ However, this discovery at that time did not catch much attention to persisters due to lack of deep understanding.

In the following 30 years, researchers conducted numerous studies to better understand persister cells and their important role in clinic.^63^ Until 1970, another category of persistence was observed by Alexandre Tomasz: a novel type of pneumococcal mutant that grows at normal generation times, is as sensitive to growth inhibition by penicillin as the wild-type parent strain, experiences only a very slow loss of viability, and does not lyse at all during exposure to penicillin.^64^ To differentiate it from the persistence found earlier (named as physiological or phenotypic tolerance many years ago), the terms “antibiotic tolerance” and “genotypic tolerance” were introduced to describe this novel type of bacterial response to antibiotic treatment.^64^ In 1974, Gary Best identified the first genotypically tolerant clinical isolate of *S. aureus* (strain Evans), which had the same oxacillin MIC as the rest of the strain but survive from high concentrations of the drug.^65^ In 1976, Mayhall et al. described a high prevalence (55%) of persisters among clinical isolates of staphylococci.^66^ In 1977, tolerant response was observed in *Streptococcus sanguis* (*S. sanguis*) by Diane Horne and Alexander Tomasz.^67^ At that time, it came to realize that persistence, which differs from previously described forms of penicillin resistance, is common and important in clinic.^68^

Since then, numerous cases of treatment failures caused by over 20 species of tolerant bacteria in humans with infections were reported,^69–77^ but there was no strong direct evidence showing that antibiotic tolerance affects the treatment of human infections. In 1983, Brennan and Durack showed a clear relationship between degree of *S. sanguis* tolerance and efficacy of treatment in the rabbit endocarditis model, in which tolerant *S. sanguis* survived better than non-tolerant bacteria after 5 days of treatment.^78^ At the same year, by repeatedly exposing growing *E. coli* to ampicillin, Harris Moyed identified *hipA* mutant with no change in MIC but had higher persistence, which was considered to be due to mutation in a gene related to the formation of persisters.^79^ These initial studies have deepened our understanding of persister/tolerant bacteria and bacterial persistence at that period.

During the 1990s-2000s, as the wide application of indwelling devices (cardiac stents, urinary tract indwelling catheters, etc.) and the increase of immunocompromised patients (cancer chemotherapy or HIV infection, etc.), the number of chronic persistent infections including biofilm infections increased dramatically.^80,81^ In 2000, Kim Lewis established a link between bacterial persistence and biofilm infections in *Pseudomonas aeruginosa* (*P. aeruginosa*).^82,83^ It was then discovered that biofilms contain persisters which are subsequently recognized as the culprit for the difficulty in curing biofilm infections, relapse after treatment, and other chronic persistent infections.^22,84^ Since then, persisters and persistent infections have garnered the interest of an increasing number of scientists worldwide.^14,28,85–90^ Nevertheless, no persister drugs that kill persister bacteria and eradicate biofilm infections exist until recently. Tuberculosis serves as a prime example of the significance of bacterial persisters during infection and drugs targeting persisters, as the unique anti-persister drug pyrazinamide (PZA) plays a crucial role in shortening TB therapy and reducing relapse rates.^17,91^

## Characteristics and detection methods of persisters

### Characteristics of persister cells and their distinctions from resistant and “tolerant bacteria”

Persisters exhibit phenotypic heterogeneity, which includes metabolic diversity, variation in persistence levels, and differences in colony sizes. (1) Metabolic diversity. The bacteria in the community have different metabolic states, including metabolic quiescence, slow metabolism, etc., that is, the individual bacterial persisters have different persistence abilities. Balaban and colleagues proposed that the non-growing (metabolically stagnant) persisters induced by external environmental factors are called type I persisters, such as those produced by culturing bacteria in liquid medium to stationary phase in vitro; The slow-growing (slow-metabolizing) persisters that are spontaneously generated by non-external factors are called type II persisters, and this group of bacteria will continue to divide and proliferate slowly and can return to normal bacteria.^25^ In fact, the metabolic heterogeneity of persisters is much more complex than that of type I and type II persisters. For example, in the group of type I or type II persisters, bacteria do not display the same metabolic states. Additionally, the metabolic state of persisters is not invariable, and it will change with the change of environmental conditions. (2) Variation in persistence level. It has been proposed that there is a hierarchy of persistence levels within persister continuum, where some persisters have strong persistence ability, which is called deep persistence, while some other persisters have weak persistence ability, which is called shallow persistence.^24,92,93^ In addition, in the studies of dormant microbes such as *Vibrio cholerae* (*V. cholerae*),^94–96^ *Legionella pneumophila*,^97–102^ *M. tuberculosis*^103,104^ etc., it has been found that persisters with deeper persistence levels can be viable but non-culturable (VBNC)^105^ but resuscitated and grow under appropriate conditions such as conditioned medium or co-culture with host cells,^106–108^ which is not covered by the conventional persister definition.^24^ (3) Differences in colony size. Heterogeneity in colony size can be reflected in the emergence of small colony variants (SCVs),^109^ which have been observed in various bacteria including *M. tuberculosis*,^110^ *S. aureus*,^111–114^ *E. coli*.^115,116^ These variants are characterized by their significantly smaller size (approximately 5 or 10 times smaller than the most common colony type) and slower growth rate compared to the parent strain. SCVs have been linked to increased antibiotic tolerance and persistence.^109,117,118^ These small colonies represent a subpopulation of persister cells with an extended bacterial lag phase,^119^ and their frequency within a bacterial population tends to increase following exposure to stressors such as acidic pH^120^ or reactive oxygen species.^121^ Thus, the heterogeneity of persisters is complex and dependent on the particular conditions under which the persisters are studied. We previously proposed a Yin-Yang model to more accurately reflect the heterogeneity and transformation of persisters,^24^ in which “Yin” and “Yang” represent persisters and growing/metabolically active bacteria, respectively. These two states of bacteria are not absolutely independent of each other, but can be interconverted to each other.^24^

Persisters are relative and highly dependent on different factors, including the type of bacterial strain, the specific antibiotic used, and the environmental conditions,^37^ such as the following: (1) The growth phase of bacteria. The percentage of persister in stationary phase was higher than that in logarithmic phase.^122–124^ For example, in the study of the formation of persisters during the growth of *E. coli* in vitro, it was found that the proportion of persisters was very low at the early stage of log phase growth, but the proportion of persisters increased rapidly at the late stage of log growth; In the stationary phase of bacteria, the proportion of persisters reached the highest and stabilized at about 1%;^125^ (2) Nutrient composition, pH and gas composition in the environment during bacterial culture can also affect persister formation. For example, amino acid limitation leads to a decrease in the growth rate of bacteria and triggers stress responses, including the inhibition of drug target function mediated by ppGpp, which renders bactericidal drugs ineffective in killing the bacteria.^126^ However, it is important to note that the tolerance induced by amino acid-limiting conditions is typically shorter in duration compared to tolerance observed during the stationary phase of bacterial growth.^126^ Furthermore, it also has been demonstrated that hydrogen sulfide (H_2_S) and nitric oxide (NO) can induce antibiotic tolerance through anti-oxidative defense.^127,128^ (3) The environment of antibiotic exposure, including the type, concentration, time and other factors of antibiotic exposure.^129,130^ Specifically, the number of persisters in the same bacterial strain can vary depending on the type of antibiotics used.^122,131–134^ For instance, drugs that reduce bacterial metabolic activity and interfere with bacterial growth and replication, such as quinolones and macrolides, will lead to more bacteria entering a persister state to resist the effects of antibiotics.^42^ Additionally, the duration and concentration of antibiotic exposure also influence the formation of persister bacteria, affecting both the number and degree of persistence.^122^ It is important to note that different bacterial species, even when exposed to the same antibiotic at the same concentration, may not produce the exact same number of persisters.^129^ Furthermore, the mechanism of persistent bacteria formation under the action of different antibiotics is also different, such as varying importance of individual persister genes in tolerance to different antibiotics.^41^

Persisters have the ability to survive in the presence of antibiotics, but they differ from resistant bacteria. Persisters have strong tolerance to antibiotics, and show multi-drug tolerance (MDT).^135^ The tolerance of persisters to antibiotics is only expressed at the phenotypic level, and there is no mutation in resistance genes as in persister bacteria, so it is different from antibiotic-resistant bacteria due to genetic mutations or antibiotic resistance genes. The minimum inhibitory concentration (MIC) of antibiotic-resistant bacteria to antibiotics is increased, while that of persisters remained unchanged or decreased. However, persisters and antibiotic-resistant bacteria are not completely unrelated, as they may interconvert and overlap as indicated in the Yin-Yang model. For instance, persisters under specific conditions can also facilitate drug-resistance gene mutations to form antibiotic-resistant bacteria, and persisters are also found in the antibiotic-resistant bacterial population.^136,137^ At present, the mechanism of tolerance of persisters to multiple antibiotics is not very clear, but the current research shows that the mechanism of tolerance of persisters to antibiotics is generally different from the mechanism of bacterial resistance,^125^ though enhanced efflux and reduced entry may be shared.

**Fig. 2:**
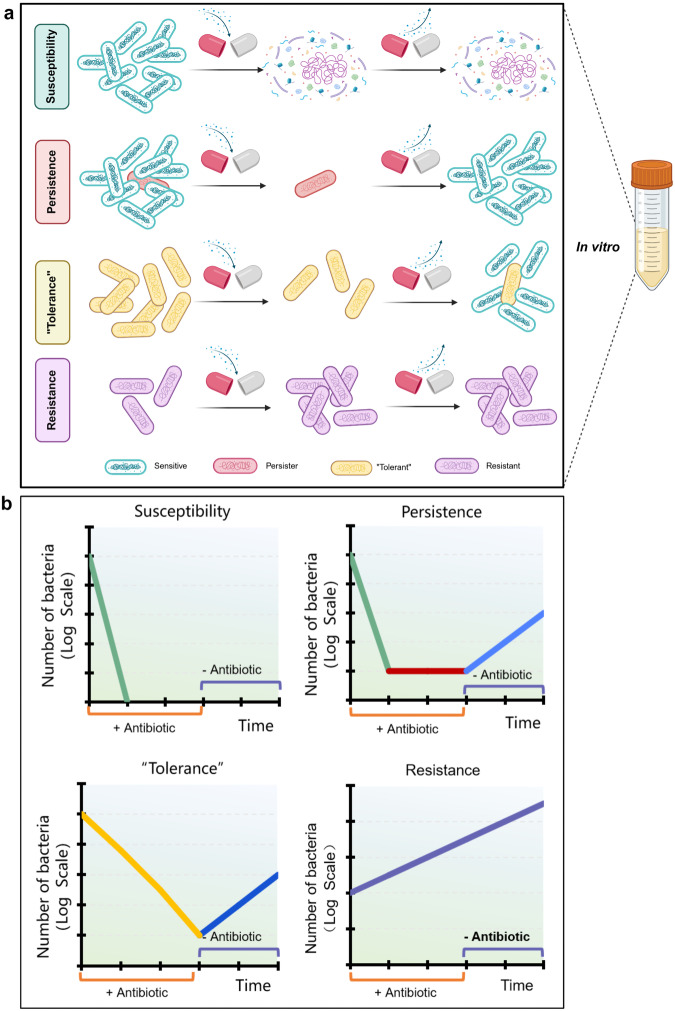
Distinguishing characteristics of persister cells from resistant and tolerant bacteria in in vitro models. According to the definition reported in the literature, when exposed to antibiotics, the homogeneous population of sensitive (green), persister (red), “tolerant,” (yellow) or resistant bacteria (purple) exhibit different scenarios (**a**) and antibiotic killing kinetics (**b**). Following the addition of a bactericidal antibiotic, sensitive bacteria could be completely killed and its kill curve is a decreasing straight line. Even after the antibiotic is removed, the bacteria cannot revive. Persister refers to a small fraction within the bacterial population that, when exposed to antibiotics, the bulk growing bacteria are killed rapidly, while the persisters are still alive. Once the antibiotic is removed, persisters can resuscitate and resume growth. “Tolerant bacteria” is a whole population of bacteria with persister-like tolerance, which are killed more slowly than normal growing bacteria and are capable of regrowth upon antibiotic removal. Resistant cells, unaffected by antibiotics, grow in the presence of the antibiotic and exhibit an ascending straight-line without being killed, indicating their survival and proliferation despite antibiotic exposure. Created with BioRender.com

In addition to persister and resistant bacteria, another term “tolerant bacteria,” has also been coined to describe bacteria that survive antibiotic treatment. As both persisters and “tolerant bacteria” exhibit the characteristic of phenotypic drug tolerance, and mechanisms associated with tolerant bacteria (such as reduced metabolism or ATP levels) have also been identified in persister,^125^ there is often confusion between the two, which puzzles many researchers.^12,138,139^ Balaban and colleagues had attempted to distinguishing between resistance, tolerance and persistence to antibiotic treatment. She proposed that persistence characterizes a bacterial subpopulation (typically less than 1%) to survive antibiotic exposure, while tolerance describes the same ability but pertains an entire bacterial population^85,137^ (Fig. 2a, b). This view distinguishes tolerance and persistence only by their penetrance within a population, but it did not define and distinguish persisters and “tolerant bacteria”. Then, Helaine and colleagues differentiated persister and “tolerant bacteria” based on their individual growth ability in the absence of antibiotic: “tolerant bacteria” are either slow-growing or non-growing, while persisters are exclusively non-growing.^140^ However, this view is inconsistent with the phenotypic heterogeneity of persisters, which suggests that persister include both (type I persisters) and slow growing bacteria (type II persisters).^25^ Additionally, Bassler and colleagues stated that persistence is another form of tolerance that is not acquired through heritable mutations, but rather through phenotypic differentiation.^86^ This claim cannot be accepted either, because mutations in genes (such as *hipA* mutant^79^) are also associated with the formation of persisters. It can be seen that how to distinguish between tolerance and persistence has remained somewhat ambiguous so far, and no one has been able to pinpoint their exact difference. We hold the opinion that there might be no fundamental difference between persisters and “tolerant bacteria” essentially but a matter of degree, and that tolerant bacteria represent shallow persisters in the heterogeneous persister continuum as proposed in the Yin-Yang model.^24^ This hypothesis can actually find answers in the research history of persisters.

Specifically, in 1970, since persistence was not well known, people mistakenly believed that “antibiotic tolerance”, refer in particular to “genotypic tolerance”, was unrelated to persistence and represented a new form of bacterial survival from antibiotic treatment. This misunderstanding led to the emergence and confusion of both tolerance and persistence in later reports. Actually, both “phenotypic tolerance” which was discovered in 1942 and named in 1986, and “genotypic tolerance” contribute to the clinical problem of bacterial “persistence”. Therefore, we believe that there is no essential difference between persisters and “tolerant bacteria” previously described in the literatures. This could explain why researchers have difficulty distinguishing between them and why they are often used interchangeably, including our own.

**Fig. 3:**
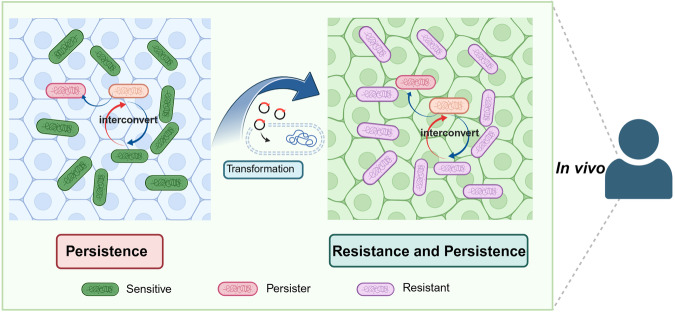
The coexistence and dynamic interconversions of sensitive bacteria, persisters/tolerant bacteria and resistant bacteria during host infections. In the host environment, the bacterial population is heterogeneous and significantly more complex than that in vitro. The metabolic activities of bacteria within the persister population are not uniform; there are bacteria with slow metabolism known as shallow persisters and those with metabolic dormancy known as deep persisters. From an evolutionary perspective, persistence under certain conditions can lead to resistance development, via mutations or transfer of resistance genes. Created with BioRender.com

During host infections, sensitive bacteria, persisters/tolerant bacteria and resistant bacteria may not exist independently, but could coexist simultaneously and undergo dynamic interconversions (Fig. 3). This might be a manifestation of the phenotypic heterogeneity of bacterial populations in the host.^141^ In the proposed evolutionary connection between persistence, tolerance, and resistance to antibiotics, the anticipated evolutionary trajectory starts with the development of antibiotic tolerance from antibiotic persistence, eventually culminating in antibiotic resistance,^142^ which is in line with our views as expressed in the Yin-Yang model. Based on the dynamic transformation process and the complex phenotypic heterogeneity of persisters, the “tolerant bacteria” described in the literature may be considered as one hierarchy of persisters. To avoid unnecessary confusion, in our review article, we refer persisters as metabolically quiscent bacteria exhibiting phenotypic tolerance or genotypic tolerance (mutations in persistence genes) to stress conditions.

### Methods for detecting persister cells

(1) Time-kill assay. Bacterial persistence refers to the ability of bacteria to survive during exposure to lethal bactericidal drugs or stress environment. The stronger the survival ability, the stronger the persistence ability. The traditional method to detect persisters is the time-kill assay, which was even employed by Hobby and Bigger in their original studies on persister cells in 1942 and 1944.^61,62^ This assay involves exposing bacteria to a lethal dose of antibiotics or stresses, followed by the quantification of the remaining viable bacteria using the colony-forming unit (CFU) counting method at different time points of antibiotic exposure. The persistence ability of bacteria can then be inferred based on the number of viable bacteria that remain after the exposure. While the time-kill assay is a commonly used method for detecting persister cells, it does have certain limitations. For instance, although it was already known that persistence is defined by a decreased rate of killing, the time-kill assay lacks criteria to determine the bacteria that survive antibiotic or stress treatment are persister cells, and may not distinguish between shallow and deep persisters. Moreover, the time-kill assay cannot be used to detect deep persisters in VBNC state as pointed out previously.^24^

(2) Minimum duration for killing 99% or 99.99% of bacteria (MDK99/ MDK99.99). The academic school represented by Nathalie Balaban and colleagues believe that tolerance is different from persistence. In 2017, Nathalie Balaban introduced a metric, MDK99, for measuring tolerance.^143^ Subsequently, in 2019, another metric, MDK 99.99, was proposed to determine the persistence capacity of bacteria.^85^ This approach appears to overcome the limitation of the absence of specific criteria in the time-kill assay, rendering it a promising method for detecting persister cells. In essence, MDK99 and MDK99.99 respectively represent the time it takes to kill 99% and 99.99% of bacteria with antibiotics. From this perspective, they reflect two levels of bacterial persistence to antibiotics, indirectly suggesting that their proposed tolerance and persistence do not have a fundamental difference.

Theoretically, both MDK99 and MDK 99.99 can be extracted from a time-kill curve. However, we have encountered challenges in applying this experimental framework to real-world scenarios. Specifically, when examining the time-dependent kill curve of *E. coli* under levofloxacin exposure, we observed that it did not follow a smooth mathematical pattern. As a result, it became difficult to accurately extract the MDK99 or 99.99 from a bacterial time-kill curve. We could only estimate the range based on the time points on the X-axes, such as between 2 and 3 (days or hours). This issue is evident in a study that utilized MDK99 assays to measure the variation in antibiotic tolerance among *M. tuberculosis* clinical isolates.^144^ It is worth noting that there are no practical experimental examples in published articles that determine bacterial persistence by calculating MDK99.99. Thus, it can be seen that the practicality of MDK assays still requires further validation.

(3) “ScanLag” and “ColTapp”. As early as 2010, Nathalie Balaban and her colleagues developed the “ScanLag” method for *E. coli*, which enables for the monitoring of a two-dimensional distribution of the lag time and growth of each colony on agar plates.^145,146^ Subsequently, Annelies Zinkernagel modified the ScanLag for the analysis of *S. aureus* lag time distributions, naming it ColTapp.^147^ Both ScanLag and ColTapp methods determine bacterial persisters based on colony and bacterial lag times, as persister cells tend to exhibit relatively long lag times and small colonies.^119^ Both the time-kill assay and the MDK99.99 method mentioned earlier provide a measurement at the population level but do not allow for the observation of individual persister cells within a bacterial population. As a result, both methods have limitations in accurately reflecting the phenotypic heterogeneity of persister cells within a bacterial population. However, the ScanLag and ColTapp methods have the ability to detect single colony growth and lag time, enabling them to measure the heterogeneity of persister cells. In addition, microfluidic device has also been used to detect individual persister cells with time-lapse microscopy.^25^

(4) Tolerance disk test (TDtest). In vitro studies have demonstrated that bacterial persistence can be induced by external environmental factors. It is important to note that bacterial persistence may also occur in patients, potentially leading to treatment failure. Therefore, in addition to assessing antibiotic susceptibility, the detection of bacterial persistence in a clinical setting is crucial for identifying effective treatment regimens. The Kirby-Bauer disk-diffusion assay has been widely utilized in clinical settings for determining bacterial antibiotic resistance. However, it is not suitable for detecting persistent bacteria. In 2016, Balaban and colleagues modified the standard disk-diffusion assay to enable the evaluation of persister tolerance levels.^148^ This modified test, known as the Tolerance Disk (TD) test, specifically targets persister cells. The TD test method consists of two steps: (1) placing an antibiotic disk on top of the agar plate, where the antibiotic diffuses into the agar, resulting in the formation of an inhibition zone around the disk for antibiotic-sensitive bacteria; (2) replacing the antibiotic disk with a glucose disk, which promotes the re-growth of bacteria and enables the detection of surviving bacteria. Although the TDtest method offers several advantages for routine clinical use, such as simplicity, low cost, and the ability to detect different levels of persistence, it does have limitations. One limitation is that it cannot provide the exact frequency of persister cells. Additionally, it is limited to detecting bacterial persistence under antibiotic exposure and cannot detect persister cells induced by other stress factors. It is important to address these limitations in future research to further enhance the utility of the TDtest in clinical settings.

(5) Replica plating tolerance isolation system (REPTIS). Inspired by the TDtest, in 2019, Matsuo and colleagues developed a replica plating tolerance isolation system called “REPTIS”, which is used to not only isolate but also calculate the frequency of tolerant cells.^149^ The “REPTIS” method also consists of two steps. In the first step, bacteria are exposed to a master agar plate containing antibiotics for 72 h of incubation. In the second step, colonies from the master plate that did not grow due to the presence of antibiotics are transferred onto a replica plate without antibiotics for another 72 h of incubation. The number of regrown bacteria indicates the presence of persister cells. Similar to the TDtest, the “REPTIS” method also has the potential to be used in clinical settings, but it is limited in its inability to detect triggered persistence induced by specific stressors. Further research is needed to address this limitation and develop methods that can effectively detect and characterize persister cells under various stress conditions in clinical settings.

(6) For VBNC persisters in non-culturable state, non-culture method could be used to evaluate if they are viable bacteria. VBNC bacteria have an intact cell membrane and can be tested with the LIVE/DEAD™ Bacterial Viability Assay.^47,150^ Reverse transcription polymerase chain reaction (RT-PCR) can also be used to detect VBNC.^151^ To detect VBNC bacteria using RT-PCR, specific marker genes or gene regions associated with the target bacteria can be selected. These marker genes may vary depending on the bacterial species of interest. For instance, using mRNAs encoding the lipopolysaccharide gene *rfbE* and the H7 flagellin gene *fliC* as the markers to detect the VBNC state of *E. coli* O157:H7;^152^ the VCA0656 gene related to the aminoimidazole riboside kinase protein was used as a genetic marker to precisely detect the VBNC state of *V. cholerae*;^151^ two selected housekeeping genes, 16S-23S rDNA and *rpoS*, proved to be good viability markers for *Vibrio parahemolyticus* VBNC state.^153^ In addition to the RT-PCR, droplet digital PCR (ddPCR) method is another suitable approach for counting the numbers of single-copy genes on the chromosome. It has emerged as a new tool for quantifying VBNC cells in recent years, demonstrating higher accuracy and sensitivity compared to qPCR.^154^

In summary, these detection methods are designed based on certain characteristics of persister cells, and each method has its own advantages and disadvantages. Among them, the TDtest method has great potential for clinical application due to its advantages in evaluating tolerance levels. However, methods such as time-kill assay, MDK99.99, and “REPTIS” are labor-intensive, which limits their application in clinic. “ScanLag” and “ColTapp” have advantages in detecting the heterogeneity of persister cells, but require special equipment (such as automated agar plate imaging as well as single-cell microscopy) and professional analysts. Therefore, research still needs to further improve and develop more comprehensive, flexible, and easy-to-operate methods to promote routine detection of persister cells in clinic.

## Persisters and chronic persistent infections

Persisters have been identified in various pathogens, including bacteria, fungi, and parasites. Among these, chronic persistent bacterial infections have been extensively studied in urinary tract infections, tuberculosis, endocarditis, refractory cystic fibrosis infections,^21,84,155,156^ as well as indwelling device-related infections like pacemaker-related infections and prosthetic joint infections.^157,158^ The presence of persister cells in these infections contributes to their chronicity and the difficulties in eradicating them with conventional antibiotic treatments. In recent years, as our understanding of persisters has grown, numerous studies have highlighted the association between persisters and treatment challenges in chronic persistent infections. These challenges include recurrent infections, bacterial resistance, and biofilm formation. The following descriptions provide further insight into these issues:

1. Recurrence of infection. Persisters play a significant role in the recurrence or relapse of infections after treatment. After bacterial infections, under the joint action of antibiotics and immune system, most bacteria die, and a small number of bacteria adjust their metabolic state and change into persisters with slow or reduced metabolism to survive. At this time, the infection symptoms may be alleviated or disappear. However, if antibiotic treatment is stopped or the immune system function of the host is impaired, the bacteria can resume growth, resulting in recurrence of infection and associated symptoms. If antibiotics are continued for a long period of time, it may still not be able to kill the persisters, while increasing the probability of drug-resistant bacteria.^22,159^
2. Bacterial resistance. Persisters serve as an evolutionary reservoir from which resistant bacteria can emerge.^160^ In the early days, researchers believed that persisters were growth arrested.^25^ At this stage, the persistence and drug resistance of bacteria were considered as independent mechanisms to resist drugs. Later, it was gradually realized that the apparent stability of persister numbers was actually due to a dynamic state of balanced death and division, rather than generally arrested growth.^161^ Furthermore, whole-genome sequencing of microbes isolated from patients who experienced recrudescent infections revealed the presence of several drug resistance mutations in antibiotic-resistant bacteria, indicating that evolution towards resistance might occur during the persistent infection.^162–164^ In 2017, Nathalie Balaban conducted in vitro evolution experiments that revealed the precedence of persister cells over resistance.^165^ These experiments showed that the bacterial cultures exhibited tolerance several cycles before the emergence of resistance. The reason behind this phenomenon may be attributed to the fact that stress response programs, crucial for the formation and survival of persister cells, can also expedite adaptive genome-wide mutagenesis.^166–169^ Another significant mechanism through which persistence contributes to antibiotic resistance is the promotion of horizontal transfer of drug resistance genes through stress responses.^170,171^ For example, the SOS response increase the frequency of horizontal gene transfer in *E. coli* and *V. cholerae*, specifically facilitating the exchange of resistance elements for aminoglycosides, lincosamides, and antifolate antibiotics.^170^ Salmonella persister cells have also been found to facilitate the dissemination of antibiotic resistance plasmids in the intestinal tract.^172^ Overall, persistence not only enables bacteria to survive antibiotic exposure but also serves as a pathway for the development of antibiotic resistance.
3. Biofilm infections. In the 2000s, researchers established a link between bacterial persistent infections and biofilms.^82^ Since then, studies on persister cells and biofilm infections have been conducted. In both *P. aeruginosa* and *S. aureus* infections, it has been observed that biofilm cells exhibit greater tolerance to antibiotics compared to planktonic cells.^83,173^ This increased tolerance is attributed to the higher number of persister cells present in the biofilm compared to planktonic cells.^174,175^ The mechanism by which bacteria within biofilms become highly tolerant to antibiotics is believed to involve the starvation-signaling stringent response,^166,175^ important for persister formation, but also physical barrier to antibiotic exposure posed by the biofilm structure. Within biofilms, nutrient availability can be limited, triggering the stringent response and promoting the formation of persister cells.^176^ The discovery that the anti-persister drug candidate ADEP4 can effectively kill persister cells by activating ClpP and subsequently eradicate chronic biofilm infections provides further confirmation of the role of persisters in biofilm-induced persistent infections.^177^ Kim Lewis proposed a model of recurrent biofilm infection which shows a good relationship between biofilm-persisters and biofilm infection recurrence.^178^ The model suggested that metabolically active bacteria and persisters coexist in biofilm infections. Antibiotic treatment can kill most of the metabolically active bacteria, and the immune system can remove part of the metabolically active bacteria and the persisters outside the biofilm, while the persisters within the biofilm continue to survive under the protection of the biofilm. When the antibiotic is removed or the concentration of the drug is reduced, the remaining persister bacteria in the biofilm continue to reproduce and re-form the biofilm infection,^24^ resulting in recurrence and persistence of the infection.

## Molecular mechanisms of persister formation and survival

Due to the complex characteristics of persisters, such as heterogeneity, relative nature, transience, small numbers, the study of the molecular mechanisms of their formation and survival is challenging. There are mainly two models for the study of genes related to persisters:

1. Screening single gene knockout or overexpression library or transposon insertion library.^179–184^ Firstly, screening for bacteria with reduced or increased persistence capacities, and then further verifying the relevant gene by PCR and sequencing. Through the gene knockout library model, the genes related to the formation of persisters are mainly global regulatory factors *phoU*,^37^ *dksA*,^181^ *dnaK*,^181^ *hupAB*^181^ and *ihfAB*,^185^ energy metabolism-related genes *sucB*^92^ and *ubiF*,^92^ etc., suggesting that the genes affecting bacterial persistence are not a single gene or a pathway-related gene.
2. Sorting or isolating persisters for transcriptome sequencing analysis. One approach is to treat bacteria with bactericidal antibiotics such as beta-lactam antibiotics, killing metabolically active bacteria and subjecting the remaining persisters to transcriptome sequencing.^124,135^ However, this method will change the external environment of the persisters after lysis of most bacteria, resulting in gene expression of the persisters being affected. The second method is to construct fluorescent bacteria containing degradable GFP controlled by ribosome promoter, and use flow cytometry or magnetic bead sorting to sort out bacteria with weak GFP fluorescence (bacteria with low translation level are considered as persisting bacteria) for transcriptome sequencing analysis.^26,186^ This method also has certain disadvantages, such as the environment and density of the bacteria are changed after the sorted persisters are transferred to a new buffer or medium. In the new environment, the persister bacteria will begin to recover, resulting in a decrease in persistence capacity. Through transcriptome sequencing, the genes related to persistence bacteria were mainly related to biosynthesis (down-regulated expression), such as *atpA*, *fdxC,* etc.,^124^ and toxin-related genes (up-regulated expression), such as *relBE*, *mazEF*, *dinJ*, *yafQ* and *ygiU*.^22^ Additionally, laboratory evolution of persistence and whole-genome sequencing has been used in the recent studies to identify genes involved in persistence.^187–189^ Through whole-genome sequencing, some new persistence related genes such as *atl* which encodes a bifunctional autolysin,^187^ *nuoAHJKLMN* which encodes NADH/ubiquinone oxidoreductase that translocates protons across the membrane,^188^ *gadC* which encodes a central component of the *E. coli* acid resistance system, and *oppB* which encodes membrane topology of the integral membrane components have been identified.^189^

Now we know the persistence mechanisms, mainly through the study of *E. coli*, *S. aureus*, *M. tuberculosis*, however, it is noteworthy that the mechanisms of persister formation in different bacteria has a certain degree of conservation, that is, with convergent evolution characteristics.^24^ Moreover, different persistence states of the same bacteria, such as stationary phase persisters, SCVs, L-form bacteria, VBNC, biofilm persisters, etc., may have similar and overlapping related persister genes and pathways.^24,190–192^ In addition, many persister genes or mechanisms exist in the same bacterial persisters and their roles are additive and redundant. Below we describe specific genes and pathways associated with persistence (Table 1).

**Table 1:** Genes and pathways involved in persister formation or survival

Pathways		Genes involved in persistence
Toxin-antitoxin module		*hipBA, relBE, mazEF, dinJ-yafQ, mqsRA, ccdAB, tisAB, yefM-yoeB, symER, yafN/yafO*
Metabolism	Energy metabolism	*sucB, ubiF, glpD, plsB, acnB, nuoI, ndh, panD, glpK, prpR, prpD, prpC*
	Protein degradation systems and trans-translation	*ssrA, smpB, clpC1, dnaK-clpB*
	Purine and amino acid metabolism	*purB, purN, purM, gltT, argJ, rpmF*
	Metabolic regulators	*phoU, IHF, arcA, Cra, cAMP receptor protein (CRP), dksA, FNR, lrp, rpoS, proQ*
DNA damage repair		*recA, lexA, umuDC, uvrAB, cspH, htrA, ibpAB, htpX, clpB, recC, ruvA, uvrD*
Stress response	SOS response	*lexA, recA, recC, ruvA and uvrD*
	RpoS (σS) -mediated response	*rpoS*
Cellular communication	Quorum sensing molecules	*oxyR, pspBC*
	Stringent response and ppGpp	*relA, spoT, raiA, rmf*
Efflux pump system		*tolC, msmK, acrA*
Epigenetic modifications and others		*dam, tacT, PNPase, ryhB*

### Toxin-antitoxin modules (TA modules)

TA modules are composed of toxin and antitoxin genes and are widely distributed in bacterial and archaeal plasmids or genomes.^193–195^ Toxins are a stable protein, which are involved in the inhibition of bacterial replication or translation. Antitoxins are unstable proteins or RNAs that inhibit the activity of toxins. When exposed to “stresses”, bacteria can make toxins work by degrading antitoxins.^196^ TA modules are classified based on the nature of the antitoxin and its mechanism of action. In the context of persister formation, the widely studied TA modules are type I and type II.^197–200^

*hipA* was the earliest persistence-related gene found in ref. ^79^ which encodes HipA toxin protein and combines with antitoxin encoded by *hipB* to form HipBA module, but its role in persistence is still inconclusive. It has been proposed that the HipA toxin works by phosphorylating the active site of glutamyl-tRNA synthetase (GltX), which leads to its inactivation.^201^ This, in turn, causes an increase in the concentration of uncharged tRNAs at the ribosomal A site, triggering RelA-dependent (p)ppGpp synthesis and inducing a high level of persistence.^201^ Some other studies have also suggested that HipA-induced persistence depends not only on (p)ppGpp but also on the 10 mRNase-encoding TA modules, polyphosphate and Lon protease.^202^ However, four years after publishing the article, the authors became aware that the previously described contribution of polyphosphate to bacterial persistence was an artifact resulting from inadvertent lysogenization with a bacteriophage.^203^ Therefore, the connection of polyphosphate and HipBA modules in persister formation is no longer supported. Recently, it has been found that HipA could mediate persistence through not only GltX but also several other targets including another aminoacyl-tRNA synthetase TrpS,^204^ the negative modulator of replication initiation SeqA, transcriptional factor RcsB, and 30S ribosomal protein RpsI.^205^ In addition to *hipBA*, several other TA modules have been associated with persister formation. These include *relBE* (toxin RelE),^206,207^ *mazEF* (toxin MazF),^208^ *dinJ-yafQ* (toxin YafQ),^209,210^ *mqsRA* (toxin MqsR),^211,212^ *ccdAB* (toxin CcdB),^213^ *tisAB* (toxin TisB),^214–216^ *yefM-yoeB* (toxin YoeB),^217^ *tacAT* (toxin TacT)^218^ and others.^219–222^ Among them, the majority belong to type II TA modules, including *hipBA*, *relBE*, *mazEF*, *dinJ-yafQ*, *tacAT* and *yefM-yoeB*;^222–224^ the remaining ones belong to type I TA modules.

**Fig. 4:**
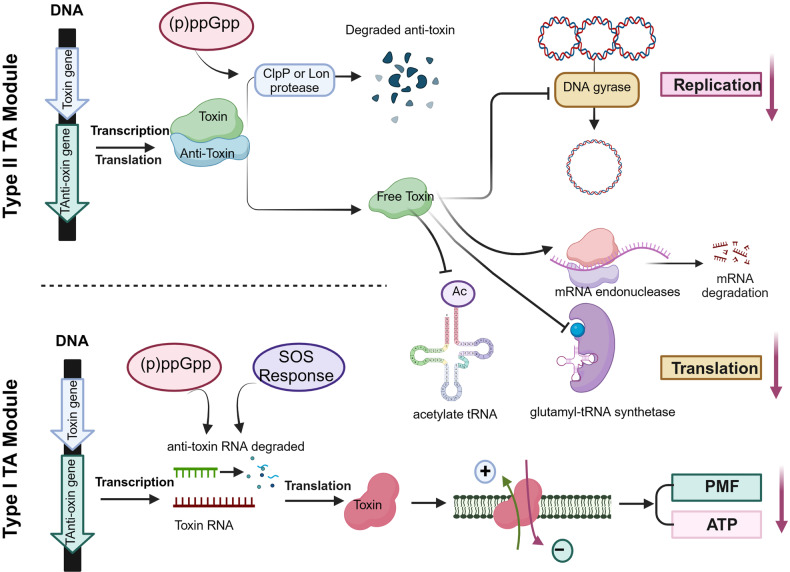
Mechanisms of persister formation via TA modules. The anti-toxins of type II TA modules are proteins that are usually degraded by the protease ClpP or Lon in response to (p)ppGpp signaling. These toxins mediate bacteria to enter a persistence state by disrupting replication and translation processes, such as interfering with DNA gyrase, acting as mRNA endonucleases, inactivating glutamyl-tRNA synthetase (GltX) and acetylate-tRNA. The anti-toxins of type I TA modules are antisense RNAs which are activated by the “SOS” response and (p)ppGpp signaling. These toxins are usually small proteins that insert and form pores in bacterial membranes, causing loss of proton motive force (PMF) and ATP production. Created with BioRender.com

The activation of TA modules often leads to the accumulation of free toxins, which can result in bacteriostatic growth inhibition and antibiotic tolerance.^225,226^ However, the activation and mechanisms of the type II and type I TA modules differ. In general, the anti-toxins of type II TA modules are proteins which are usually degraded by the protease ClpP or Lon in response to (p)ppGpp signaling.^208,223,224,227,228^ The anti-toxins of type I TA modules are antisense RNAs which are activated by the “SOS” response and (p)ppGpp signaling.^215,225^ Type II toxins mediate bacteria to enter a persistence state through various mechanisms,^229–232^ as illustrated in Fig. 4. Primarily, these toxins primarily act as inhibitors of replication and translation.^233^ They interfere with DNA gyrase, leading to the inhibition of DNA replication (e.g., HipA). Moreover, they function as mRNA endonucleases, either ribosome-dependent (e.g., RelE family) or ribosome-independent (e.g., MazF family),^197,199^ consequently disrupting the translation process (Fig. 4). Additionally, certain type II toxins, such as HipA^201^ and TacT,^218,234,235^ have been found to inactivate glutamyl-tRNA synthetase (GltX) or acetylate tRNA respectively. Type I toxins are usually small proteins, such as TisB and HokB, that insert and form pores in bacterial membranes, causing loss of proton motive force (PMF) and ATP production^236^ (Fig. 4). Thus, the activation of TA modules and the accumulation of toxins have been implicated in persister formation,^124,237,238^ a state in which a subpopulation of bacteria becomes dormant and exhibits increased tolerance to antibiotics or other stresses.^26^

Research findings have demonstrated that over-expression of toxin genes can indeed enhance bacterial persistence.^135,239–241^ However, the knockout of cumulative deletion of 10 TA modules or more decreases tolerance to antibiotics, but single deletions had no effect on persister levels,^226,242^ which may be due to the abundance of TA elements in bacteria. For example, *E. coli* has at least 15 TA modules and *M. tuberculosis* has at least 80 TA modules, which are highly expressed in bacteria exposed to stress factors. Knockout of one of these genes is compensated by other genes, and the persistence of bacteria is reduced when multiple genes are knocked out simultaneously.^243^ Due to their redundancy, though multiple TA systems have been found to be involved in bacterial persistence, developing novel anti-persister drugs targeting these systems may be challenging. However, recent research has shown that inhibiting toxin HipA can interfere with persister formation.^244^ This finding suggests that interfering with toxin-antitoxin modules may have therapeutic implications for treating bacterial infections. Finally, it is worth mentioning that bacteria such as *B. burgdorferi* and *S. aureus* had persisters but *B. burgdorferi* has no TA modules and *S. aureus* has TA modules but do not play a role in persistence,^48^ suggesting that TA modules are not the main determinant of bacterial persistence, despite TA was first identified to be involved in persistence phenomenon.^79^ And further studies are still needed to uncover the precise roles of different TA modules in different bacterial persistence.

### Metabolism and metabolic regulation

#### Energy metabolism

**Fig. 5:**
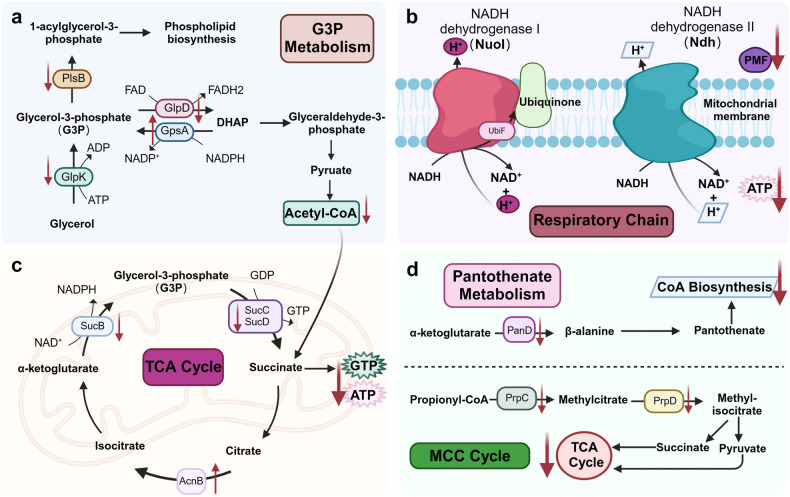
Mechanisms of persister formation via energy metabolism. Persisters have lower metabolic activity than non-persisters and this reduced energy metabolic activity could be mediated through (**a**) G3P metabolism, (**b**) aerobic respiration, (**c**) tricarboxylic acid (TCA) cycle and (**d**) methylcitrate cycle (MCC), which may allow persisters to enter a dormant state and survive in adverse conditions. For example, reduced ATP levels decrease the activity of ATP-dependent antibiotic targets, while decreased proton motive force (PMF) restricts the entrance of antibiotics such as aminoglycosides and thus enhances the tolerance ability. Created with BioRender.com

Persisters are dormant and the metabolic process is obviously slowed down or stopped,^245^ and thus genes that alter cell metabolism are involved in the formation of persisters, such as *sucB* encoding the E2 subunit of the α-ketoglutarate dehydrogenase complex,^92^ *sucC* and *sucD* encoding succinyl coenzyme A (succinyl-CoA) synthetase^242^ in the tricarboxylic acid (TCA) cycle (Fig. 5). In the TCA cycle, both the α-ketoglutarate dehydrogenase complex and succinyl-CoA synthetase play critical roles in cellular metabolism, particularly in the generation of energy. Specifically, α-ketoglutarate dehydrogenase catalyzes the dehydrogenase of α-ketoglutarate to succinyl-CoA (2-oxoglutarate + NAD^+^ + CoA-SH → Succinyl-CoA + NADH + H^+^ + CO_2_); succinyl-CoA synthetase breaks down succinyl-CoA into succinate and CoA (Succinyl-CoA+ GDP+ Pi → Succinate + GTP + CoA). We and others have previously found that bacterial persistence levels decreased when genes (*sucB*, *sucC*, and *sucD*) encoding the relevant enzymes in the catalytic α-ketoglutarate to succinate reaction pathway are disrupted.^92,242^ In contrast, disruption of *acnB* gene encoding aconitase, which catalyzes the conversion of citrate to isocitrate, improved the bacterial persistence ability.^242^

Changes in the respiratory chain activity can also cause alterations in bacterial persistence levels (Fig. 5). In the respiratory chain, ubiquinone forms hydroquinone upon acceptance of 2e^-^ and 2H^+^ from the cytosol, which plays a key role in ATP production and maintenance of membrane potential. *ubiF* is the key gene for ubiquinone formation in the aerobic biosynthetic pathway.^246^ Compared with the wild strain, the knockout strain of *ubiF* is more sensitive to antibiotics and the persistence level is correspondingly reduced.^92,247^ Aerobic respiration in bacteria is mediated by NADH. The inactivation of NADH dehydrogenase I (*nuoI*) and NADH dehydrogenase II (*ndh*) reduces oxidized NAD (NAD + ), proton motive force (PMF), and positively charged aminoglycosides are difficult to enter cells, thus inhibiting the killing effect of aminoglycosides on bacteria, that is, improving the persistence capacity of bacteria.^248^ After *S. aureus* randomly enters the stationary phase, the activity of ATP-dependent antibiotic targets (DNA gyrase, DNA topoisomerase, RNA polymerase, etc.) decreases with the decrease of ATP, and the bacteria can only maintain limited physiological activities, and the persistence ability is enhanced.^249^ Therefore, the intracellular ATP level also affects the formation of persisters.

During the process of glycerol-3-phosphate metabolism in *E. coli*, glycerol kinase GlpK catalyzes the conversion of glycerol to glycerol-3-phosphate (G3P), the G3P acetyltransferase PlsB catalyzes the first step of phospholipid synthesis (G3P → 1 acyl G3P), and G3P dehydrogenase GlpD converts G3P to DHAP under aerobic conditions (Fig. 5). It has been reported that the overexpression of GlpD in *E. coli* increased the bacterial tolerance to ampicillin and ofloxacin, while a *glpD* deletion mutant had a decreased level of persisters in the stationary phase.^250^ The persistence ability of *plsB* mutant was 100–1000 times lower than that of wild strain, but the mRNA of *plsB* gene in persisters was not changed compared with that of metabolically active bacteria in the wild-type strain,^250^ suggesting that *plsB* may be a gene related to the entry of bacteria into persistence state, but not related to maintenance of bacteria in the persistence state. Glycerol kinase (GlpK) catalyzes the conversion of glycerol to glycerol-3-phosphate, allowing it to enter the process of glucose metabolism. In our previous studies, we found that *glpK* was involved in persister, L-form and biofilm formation in *S. aureus*.^43^ A recent study has found that the *glpK* of *M. tuberculosis* is associated with the tolerance of the bacterium to anti-tuberculosis drugs,^251^ which further suggests the relationship between GlpK and the formation of persisters and stress survival.

Aspartate decarboxylase (encoded by *panD*) catalyzes L-aspartate to β-alanine, which is one of the key enzymes in pantothenate metabolism and acetyl-CoA synthesis, and is essential for energy metabolism and fatty acid metabolism (Fig. 5). Our previous studies found that aspartate decarboxylase PanD is a target of pyrazinamide (PZA), which can explain the mechanism of PZA in killing persisters by inhibiting PanD to affect acetyl-CoA synthesis and energy metabolism of persisters.^31,252,253^ In addition, in the study of *M. tuberculosis*, it was also found that the transcription factor *prpR* of the *prpDC* operon, which is involved in energy metabolism, is also involved in persistence.^254^ The proteins encoded by *prpD* and *prpC* catalyze the first two steps of the methylcitrate cycle (MCC), converting propionyl-CoA to pyruvate, while *prpR* is a transcription factor of the operon, so *prpR* mutations affect the energy metabolism and persistence capacity of *M. tuberculosis*.

#### Trans-translation and protein degradation systems

Trans-translation exists in almost all bacteria, and plays an important role in rescuing stranded ribosomes and degrading toxic proteins during protein translation.^255–258^ It is required by diverse bacteria, especially in response to stresses.^259,260^ In our previous studies, we discovered that one of the mechanisms of action of PZA, an anti-persister drug, is to inhibit trans-translation and protein degradation by binding to bacterial ribosomal protein S1 (RpsA), suggesting that trans-translation and protein degradation is involved in persistence.^29^ Indeed, subsequent studies with mutant strains defective in trans-translation component *ssrA* and *smpB* in *E. coli*, showed that the persistence ability of the mutants to diverse antibiotics and stresses decreased significantly, which further confirms the roles of trans-translation and protein degradation in bacterial persistence.^36^

The finely tuned Clp protease system plays a crucial role in bacterial survival under stress conditions.^261–263^ During this process, damaged proteins are identified as substrates and a variety of major stress regulators are controlled by Clp-mediated proteolysis.^264^ A functional Clp protease complex consists of an ATP-consuming hexameric Clp-ATPase and the barrel-shaped proteolytic core ClpP. Usually, bacteria have various Clp-ATPases, including ClpC, ClpX, ClpA, or ClpE, that interact with the same proteolytic core.^264,265^ It has been found that mutations in the gene *clpC* or *clpP* are associated with PZA resistance in *M. tuberculosis*^266,267^ and persister formation in *S. aureus*,^268,269^ which verify the role of protein degradation in bacterial persistence. Furthermore, ATP depletion is the main force driving the formation of protein aggregation, and protein aggregation could be used as an indicator of dormancy depth.^93^ DnaK-ClpB can promote the degradation of protein aggregates, and interfering with the function of this gene will affect the recovery of persisters.^93^ These results further indicate the correlation between protein degradation and bacterial persistence.

Acyldepsipeptide antibiotic (ADEP4) can activate the ClpP protease, which can result in non-specific protease activity and subsequent degradation of over 400 proteins. These degraded proteins included ribosomal proteins, such as proteins S21, L9, S1 and ribosomal recycling factor, as well as FtsZ and proteins from various functional types such as purine metabolism, glycolysis, and aminoacyl-tRNA biosynthesis.^177^ Furthermore, ClpP is crucial for the proteolytic regulation of cellular levels of the MazE and RelB in type II toxin-antitoxin systems in *Streptococcus mutans*,^228,269,270^ suggesting that it is involved in the degradation of antitoxins. This may be the mechanisms through which ClpP is involved in bacterial persistence. Combining ADEP4 with rifampicin was shown to produce complete eradication of *S. aureus* biofilm persisters both in vitro and in vivo.^177^ The anti-persister effects of PZA targeting ClpC1 as well as ADEP4 targeting ClpP suggest that protein degradation pathway may serve as excellent therapeutic targets of anti-persister drug development.^271^

#### Metabolism of purines and amino acids

Purines are essential components of DNA, RNA, and ATP, and their metabolisms play crucial roles in various cellular processes. Disruption of the purine metabolism in *S. aureus*,^272^ *B. subtilis*,^273^ *A. veronii*,^274^and *V. splendidus*,^275^ influened persister cell formation. Moreover, it has been found that purine is crucial for persistence of *E. faecalis* in vivo, such as urinary tract and wound infections.^276^ Thus, it can be seen that the metabolism of purine has been linked to bacterial persister formation. Through screening a gene mutation library, we discovered that purine metabolism-related genes *purB, purM* and *purN* are involved in *S. aureus* persistence to antibiotics and stresses.^277,278^ Additionally, microarray analyses of persistent methicillin-resistant *S. aureus* (MRSA) bacteria showed that the transcription of multiple purine metabolism genes, including *purF*, *purM*, and *purN*, were significantly higher compared to non-persistent bacteria.^272^

The mechanism of purine metabolism in bacterial persistence encompasses various aspects, including energy metabolism, protein aggresome formation and intracellular efflux mechanisms.^275^ For instance, a decrease in purine metabolism has been linked to reduced energy production, such as ATP,^275^ which has been suggested to affect bacterial persistence to antibiotics by lowering the activity of ATP-dependent antibiotic targets.^186,249,279^ Additionally, the decrease in purine metabolism has been associated with protein aggresome formation,^275^ which has been reported to be linked to persister cells, as described earlier. Furthermore, inhibition of purine metabolism has been found to decrease membrane potential,^275^ which results in lower intracellular antibiotic concentrations compared to active cells, leading to enhanced bacterial survival under antibiotic treatment.^280^ Purine nucleosides, such as adenosine, guanosine, and inosine, have also been demonstrated to play significant roles in bacterial persistence.^281,282^ Adenosine and guanosine have been shown to restore susceptibility of *V. splendidus* persister cells to tetracycline.^281^ Inosine, on the other hand, has been found to modulate membrane permeability by upregulating the expression of OmpF.^283^ Inosine activates CpxA, which dephosphorylates CpxR-P and subsequently promotes the transcription of *ompF*,^284^ thereby increasing the uptake of antimicrobial molecule. All of these findings are consistent with the notion that purine metabolism is inversely correlated with bacterial persistence.

Amino acid metabolism plays a crucial role in the growth and survival of bacteria, including persistent bacteria. In persistent bacteria, amino acid metabolism is often altered to adapt to the stressful conditions they encounter. For example, persistent bacteria can exhibit changes in amino acid transport systems, allowing them to acquire amino acids more efficiently from their surroundings.^285–287^ In this discussion, we specifically focus on the role and mechanism of glutamate and arginine in bacterial persistence. Glutamate plays a crucial role in various metabolic processes in bacterial cells. Among the L-glutamate transport systems, GadC was initially identified as a regulator of acid tolerance in *E. coli*.^285^ In our previous research, we discovered that genes related to glutamate metabolism, such as *gltT*, as well as glutamate transporter genes *gadC, gltS, gltP* and *gltI* are all involved in bacterial persistence.^286,287^ Our findings demonstrated that the deletion *gltS*, *gltP* and *gltI*, resulted in decreased tolerance to various stresses, including antibiotics, acidic pH, hyperosmosis, and heat, in both *E. coli* and uropathogenic *E. coli* strains.^286^ Additionally, it also has been reported that global regulator Fis was beneficial for persister formation in *S. typhi* by repressing the gene expression associated with glutamate transport.^288^ Antibiotic tolerant *E. coli* cells upregulates glutamate decarboxylases (GadA) to counteract its intracellular acidification.^289^ These further supports the important role of glutamate metabolism in bacterial persistence.^278,286,287^

Glutamate may mediate tolerance to antibiotics and stresses through several potential mechanisms. Firstly, the glutamate-dependent acid resistance system is considered the most effective defense mechanism in safeguarding cells against low pH environments, providing a crucial protective role.^290–292^ Secondly, glutamate and its metabolite GABA serve as prominent compatible solutes in bacteria, enhancing enzyme functionality and protecting cells from various stresses such as high temperatures, freeze-thaw treatments, and drying.^293^ Thirdly, glutamate dehydrogenase catalyzes the conversion of glutamate to α-ketoglutaric acid (α-KG) through oxidation and deamination, which enables glutamate to enter the energy metabolism pathway via the TCA cycle, thereby facilitating bacterial tolerance.^287^

Besides glutamate, arginine has also been implicated in bacterial persistence. Through a comprehensive genetic screen targeting the clinically relevant strain USA300, we have identified the importance of the arginine metabolism gene *argJ* in persister formation, survival, and virulence in mice and *C. elegans*.^46,278,287^ Notably, mutations in the active site of the ArgJ protein resulted in a persistence defect, which was restored by genetic complementation and arginine supplementation in the *argJ* mutant.^46^ Furthermore, quantitative RT-PCR analysis revealed the upregulation of genes within the *arg* operon under drug-stressed conditions and in stationary phase cultures. Despite the identified role of the arginine biosynthesis pathway in persistence, the underlying molecular mechanisms remain unclear. One proposed mechanism is that the downstream products of arginine production, such as ammonia, mitigate hydroxyl radicals generated during antibiotic action that promote cell death and neutralize the acidic environment.^294^ Additionally, ornithine and polyamines have been shown to enhance cell fitness and survival against reactive oxygen species.^295^

#### Metabolic regulators

Cell metabolism is closely related to the formation and survival of persisters, so metabolic regulators are also associated with bacterial persistence, such as *phoU*. *phoU* is located on the *pstSCAB* operon of phosphate transport system and has negative regulation effect on phosphate metabolism in *E. coli*.^296^ Through screening the transposon mutant library of *E. coli*, we found that the *phoU* mutant had significant defect in persistence to various drugs and stress conditions (nutritional deficiency, high temperature, acidity, etc.), while the metabolic activity of the *phoU* mutant was higher than that of the parent strain (high expression of energy metabolism-related genes, phosphate metabolism-related genes and flagellum synthesis-related genes).^37^ These results suggest that PhoU may be a negative global regulator, mediating the persistence of bacteria by down-regulating their metabolic activities. The effect of PhoU on bacterial persistence was further validated in different bacterial species such as *M. tuberculosis*, *S. aureus* and *P. aeruginosa*. In the study of *M. tuberculosis*, we and others found that the persistence of *M. tuberculosis* in vitro and in vivo was significantly reduced after mutation of phoY proteins, a *phoU* homolog.^33,297^ In *S. aureus*, *phoU* mutation caused up-regulation of genes related to carbon metabolism and pyruvate metabolism, down-regulation of virulence genes and virulence regulatory genes,^298^ which further verified the mechanism of PhoU as a global regulator mediating bacterial persistence.

In addition, other global regulators have also been found to be involved in bacterial persistence. Lewis et al. evaluated the correlation between global regulator gene knockout strains and their persistence ability in *E. coli* and found that ATP levels increased and persistence levels decreased after integration host factor (*ihf*) gene knockout.^185^ Brynildsen *et al*. evaluated the role of seven global transcriptional regulators, ArcA, Cra, cAMP receptor protein[CRP], DksA, FNR, Lrp, and RpoS, in bacterial persistence, and found that cAMP/CRP played a central role.^299^ Recently, the RNA-binding protein ProQ has been found to be another global regulator of gene expression and contributes to persister formation in Salmonella by activating energy-consuming cellular processes.^300^

### Stress responses mediated by SOS and sigma factor RpoS

**Fig. 6:**
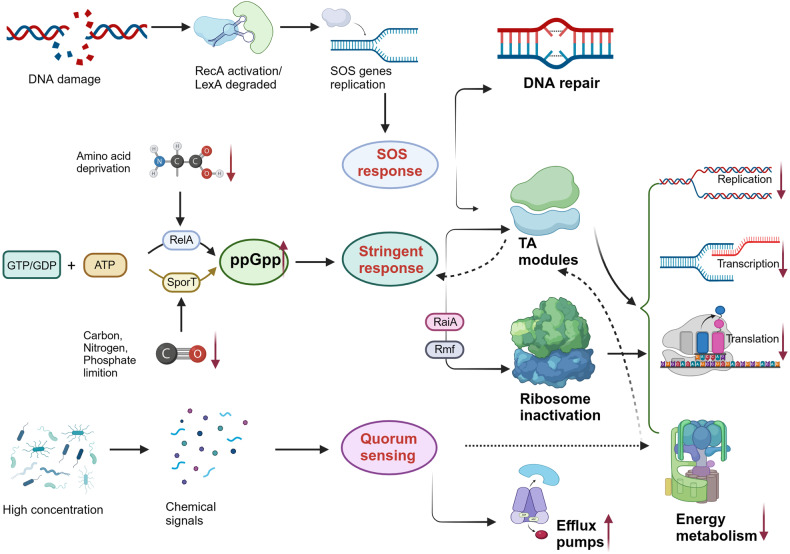
Mechanisms of persister formation via SOS response, stringent response and quorum sensing. The SOS response is triggered by DNA damage, facilitating genetic repair to enhance cell survival under stress. It also contributes to the activation of type I TA module TisB/IstR as well. The stringent response is a global response to nutrition deprivation or limitation (such as carbon, amino acid nitrogen and phosphate), where (p)ppGpp is an important messenger molecule. This response induces bacterial dormancy through downstream pathways involving TA modules and ribosomes. Quorum sensing is a bacterial communication process that coordinates behavior based on population density. Bacteria release signaling molecules (AHL, CSP, indole etc.), which can enhance efflux pumps or disrupt metabolism. Created with BioRender.com

The SOS response is triggered by DNA damage, allowing repair of genetic material to enhance cell survival.^301^ The proteins that make up the SOS system include a transcriptional repressor called LexA and a DNA-binding activating protein, RecA. After *recA* gene knockout, the persistence ability of *E. coli* to ciprofloxacin was significantly decreased, but it had no effect on the persistence to penicillin and gentamicin. The main reason is that quinolones cause bacterial DNA breaks,^134^ which activate the SOS initiation repair mechanism and related genes. This phenomenon showed that the same bacteria, under the action of different antibiotics, the genes related to the formation of persisters will have certain differences. By screening the single-gene knockout library of *E. coli*, we found that the mutations in other genes related to SOS response, *recC*, *ruvA* and *uvrD*, also cause defective persistence under the induction of rifampicin and tetracycline.^42^ Moreover, activation of the SOS response increases the frequency of SCVs, a special type of persisters, in *S. aureus*.^302^ These findings indicate that SOS response and DNA repair participate in the formation and survival of persisters (Fig. 6). In addition to DNA repair, the SOS response contributes to activating the type I TA module TisB/IstR as well.^215^ This leads to strong transcription of *tisB*, which forms ion channels in the plasma membrane, reducing the proton motive force and ATP formation, ultimately resulting in the formation of persisters.^236,303^ The induction of SOS response can also enhance the expression of fibronectin-binding protein, promote the attachment to host cells and accelerate the formation of biofilm, and improve the viability of bacteria in extreme conditions.^304^ Moreover, the SOS system (RecA), which is crucial for the development and survival of persisters, has been discovered to promote horizontal gene transfer between *V. cholerae*, thereby enhancing antibiotic resistance.^170^

RpoS (σ^S^)-mediated response can be induced by several general stresses,^305^ including nutrient deprivation, extreme pH, temperature and oxidative stress, which is associated with bacterial survival in stressful conditions as in stationary phase.^174,306^ However, its specific functions and regulatory mechanisms of RpoS in the process of persister formation are still uncertain. Some studies have found that SOS response increases tolerance to antibiotics in *P. aeruginosa*^306^ and *E. coli*.^307^ On the contrary, certain studies have demonstrated that the deletion of *rpoS* significantly enhances persistence through the upregulation of the MqsR toxin, indicating that bacteria with impaired general stress response are more prone to generating persister cells.^308^ Moreover, it has been reported that the antitoxins DinJ of the YafQ/DinJ TA module and MqsA of the MqsR/MqsA TA module have the ability to repress *rpoS* transcription and translation.^309,310^ These findings suggest that RpoS response and TA systems interact in the process of bacterial persistence. However, a recent study has revealed that RpoS had minimal impact on antibiotic persistence in *E. coli*.^311^ Therefore, further studies are required to address these discrepancies.

### Cell communication

#### Quorum sensing and signaling molecules

Bacteria can communicate with each other through chemical signals to produce phenotypic heterogeneity, which is called quorum sensing (QS).^312,313^ Many significant bacterial pathogens, such as *P. aeruginosa*, *S. aureus*, *S. typhimurium* and *V. cholerae*, employ QS cell communication to regulate the expression of numerous virulence factors and related behaviors.^314^ In 2010, Nina Möker first reported the connection between the intraspecies quorum-sensing regulatory pathway and the formation of persisters.^315^ She discovered that the quorum-sensing-related signaling molecules, phenazine pyocyanin and the acyl-homoserine lactone (AHL) 3-OC12-HSL, notably elevated the number of persisters in logarithmic *P. aeruginosa*. In 2012, Leung and Lévesque reported that another chemical signal, competence-stimulating peptide (CSP), can induce the formation of *Streptococcus mutans* persisters.^316^ This finding further supports the hypothesis that quorum sensing is implicated in bacterial persistence. Following that, the involvement of quorum sensing in bacterial persistence has been confirmed in a range of other pathogens, including *E. coli*,^317,318^ *V. cholerae*,^319^ *S. aureus*^320^ and others.^314,321^ It is possible that the impact of quorum sensing on the formation of persister cells is linked to its capacity to regulate the secretion systems (T3SS and T6SS)^322–324^ and toxin like pyocyanin.^325^ Nevertheless, additional research is needed to elucidate these connections.

The QS systems identified in *E. coli* consist of the LuxR homolog, LuxS, autoinducer-2 (AI-2) and autoinducer-3 (AI-3) systems, as well as an indole-mediated signaling system.^314^ Indole-mediated signaling is widely distributed between cells and has been shown to be involved in the formation of *E. coli* persisters.^317^ It has been reported that indole can be sensed by a population of cells in a heterogeneous manner, activate oxidative stress and phage shock pathways through periplasmic or membrane components, induce high expression of *oxyR*, *pspBC* genes, and thus contribute to the generation of persister subpopulation.^317^ This result has been confirmed in another study.^326^ Furthermore, the intestinal pathogen *Salmonella typhimurium* has also been observed to increase its antibiotic tolerance in response to indole.^327^ These findings highlight the complex interactions between indole and bacterial antibiotic tolerance. Additionally, in *E. coli*, indole still has been found to induce the expression of several multidrug efflux pumps.^328,329^ These research findings have established the relationship between quorum sensing, reactive oxygen species (ROS), and efflux pumps (Fig. 6), suggesting that quorum sensing does not act alone in mediating bacterial persistence.

However, it is important to note that previous studies have reported contrasting effects of indole on antibiotic persistence. For example, indole has been shown to decrease antibiotic tolerance of *E. coli*.^209,330^ Additionally, indole derivatives have demonstrated the ability to enhance the efficacy of aminoglycosides against stationary-phase Gram-positive bacteria under hypotonic conditions. However, these derivatives have also been found to suppress the effects of aminoglycosides against Gram-positive bacteria in the exponential-phase stage and both stages of Gram-negative bacteria.^331^ Furthermore, indole has been observed to exhibit opposite effects on antibiotic resistance, as it has been shown to reverse antibiotic resistance in *Lysobacter enzymogenes*.^332,333^ These diverse effects of indole on antibiotic persistence and resistance highlight the complexity of its interactions with bacteria. It is important to further investigate the underlying mechanisms behind these opposing effects of indole on antibiotic persistence and resistance.

#### Stringent response and guanosine pentaphosphate/tetraphosphate (pppGpp/ppGpp)

Stringent response in bacteria is a global response to amino acid deprivation or carbon, nitrogen and phosphate limitation,^334,335^ where (p)ppGpp is an important messenger molecule in this process, responsible for sensing environmental stress and inducing downstream pathways to drive bacteria into dormancy.^28,336^ The (p)ppGpp network comprises Rel/SpoT homolog (RSH) proteins with a nucleotidyl-transferase domain.^337^ In *E. coli*, the (p)ppGpp synthetase RelA is activated by amino acid starvation and heat shock, while the synthetase/hydrolase enzyme SpoT is activated by carbon, nitrogen, phosphate, iron, and fatty acid starvation.^338^ When bacteria are exposed to these stresses, the concentration of (p)ppGpp increases and acts as an alarm to coordinate many concentration-dependent process reprogramming, such as replication, transcription, and translation^339^ (Fig. 6). Interestingly, diverse bacteria deficient in (p)ppGpp production usually display massive defects in persister formation and survival.^126,166,340–345^ Also, clinical *S. aureus* mutations that partially activate the stringent response confer multidrug tolerance.^346^

The ppGpp ribosome dimerization has been proposed as a model of persister formation where ppGpp may regulate bacterial persistence levels by affecting ribosome function^347,348^ (Fig. 6). This model suggests that stress factors such as nutritional deficiency, hyperosmolality and acidity can induce the production of ppGpp and cAMP through RelA/SpoT, and ppGpp and cAMP further induce the expression of ribosome-associated inhibitor A (*raiA*) (which binds to the 70S ribosomes to inactivate it) and ribosome modulation factor (*rmf*) (which converts active 70S ribosomes into inactive 100S ribosomes through inactive 90S dimer complex). Both can cause protein synthesis disorders by inactivating ribosomes.^347^ Wood et al. previously found that after ribosome function inactivation, persister cell population in the exponential growth stage increased nearly 10,000 times.^279^ This result further confirmed the mechanism of ppGpp involved in the formation of persisters by regulating ribosome function. Additionally, since both type I and type II TA modules are activated by the (p)ppGpp signaling, it suggests that the mechanism of ppGpp involved in the formation of persisters might be through TA modules. However, this mechanism, following the retraction of a closely related previously published article titled “(p)ppGpp Controls Bacterial Persistence by Stochastic Induction of Toxin-Antitoxin Activity”,^227^ still requires further validation.

### Efflux pumps/transport systems

In addition to “passive defense” through dormancy, persisters can also excrete drugs to resist antibiotic attack by enhancing efflux activity, a form of “active defense”.^349–351^ Efflux pumps are a type of membrane protein that can actively pump drugs out of bacterial cells, reducing the concentration of antibiotics inside the cells and thus reducing their effectiveness.^352–354^ In 2016, Bai and colleagues from Peking University in China discovered that several multi-drug efflux genes, with a focus on the central component *tolC*, exhibited elevated expression levels in *E. coli* persisters.^355^ Moreover, through time-lapse imaging and mutagenesis studies, they further confirmed a direct positive correlation between *tolC* expression and bacterial persistence.^355^ This study garnered significant attention at that time,^349–351,356^ because it represented the first instance of an active mechanism contributing to stochastically induced multiple-drug tolerance. Subsequent studies further demonstrated that enhanced efflux activity contributes to the formation of persister cells.^357,358^ For example, when penicillin-exposed persisters of *S. pyogenes* (Group A streptococcus-GAS) were compared with susceptible strains, a significant number of efflux pump-related genes were transcriptionally upregulated, including at least a 4-fold increase in Resistance-Nodulation-cell Division (RND) family efflux pump-related operon genes and a 2.2-fold increase in gene expression of a homolog of the polysaccharide transporting ATP-binding protein MsmK.^359^ In addition, loss of the AcrAB efflux pump in *Aeromonas veronii (A. veronii)* decreased the formation of persisters when treated with chloramphenicol, tetracycline, fluoroquinolone and β-lactam antibiotics.^360^ Moreover, AcrR, the repressor of the AcrAB efflux pump, was also found to play a role in regulating persister formation by repressing the activity of the AcrAB efflux pump in *A. veronii*.^360^ The involvement of efflux pumps in bacterial persistence had been validated by proteomic analysis^361^ and in ex vivo models as well.^362^ The mechanism might be that efflux pumps with high activity can reduce the concentration of antibiotics in persisters, which is of great significance to the survival of the persisters.

As is well known, the efflux system is also a crucial control element for antibiotic-resistance.^352,363–366^ However, the triggers and outcomes of efflux pump changes might differ between resistant and persistent bacteria. In the case of resistant bacteria, the efflux pumps are often triggered by low concentrations of antibiotics. When antibiotics are present at levels below their effective killing threshold, as can occur with the unregulated use of antibiotics in clinical settings, bacteria can detect and respond to these low levels by increasing the expression of efflux transporters and other mechanisms through genetic alterations.^364^ This leads to the development of a more robust defense mechanism that enables bacteria to withstand higher concentrations of antibiotics that would typically be lethal to them. On the other hand, by tracking bacteria using the FlAsH-labeled TolC, researchers found that persister cells could emerge from a subpopulation that had increased levels of TolC even before treatment with the antibiotic.^355^ This suggested that in persistent bacteria, the efflux pumps are triggered by not only antibiotics but also by other factors in environment, like metabolic toxic compounds. Although persister cells enter a dormant or slow-growing state, they are not completely metabolically inactive and may produce toxic compounds that also need to be expelled.^364^ This important result also raises the question of whether the mechanism of bacterial persistence mediated by efflux pumps is independent of other mechanisms that have already been discovered.^350^ Further detailed and mechanistic investigations are essential to answer this question.

Moreover, as a recent study demonstrated the role of the AcrAB-TolC multidrug efflux pump on drug resistance acquisition by plasmid transferring,^363^ suggesting that the mechanism of bacterial resistance mediated by efflux pumps is probably not primarily through antibiotic efflux. It appears that when bacteria rely on efflux pumps as their primary defense against antibiotics and only pump out intracellular levels of antibiotics, persistence is formed. In the proposed evolutionary connection between persistence and resistance to antibiotics, the expected evolutionary trajectory begins with the emergence of antibiotic persistence, ultimately leading to antibiotic resistance.^142^ From this perspective of the mechanism involving efflux pumps in bacterial persistence and resistance, such an evolutionary trajectory seems reasonable.

### Epigenetic modifications, RNA degradation, and small non-coding RNA

Strain with deletion in adenine methyltransferase-encoding gene *dam* (*Δdam*) in uropathogenic *E. coli* (UPEC) had a significant defect in the formation of persisters, while strain with deletion in cytosine methyltransferase-encoding gene *dcm* (*Δdcm*) did not affect the formation of persisters, suggesting that adenine methylation but not cytosine methylation is involved in persister formation or survival.^367^ Dam could mediate the formation of persisters by regulating the transcription of related genes, cell movement, DNA repair and other processes.^367^ In addition to this, under stress, cellular DNA methylation status regulates global gene expression patterns to promote bacterial dormancy, when persisters recover from dormancy, some of the epigenetic traits that promote persistence can be retained in a few cells as heritable “memories” that drive evolutionary pathways over time, thereby increasing the frequency of persister formation or survival.^368^

RNA methylation has also been found to play a role in the formation of persisters. For example, methylation of the G37 site of tRNA m^1^ (at the 37th guanosine N1site at the 3’ end of the tRNA anticodon) in Gram-negative bacteria can maintain a unique double-membrane structure in its envelope, which is closely related to drug resistance and persistence in Gram-negative bacteria.^369^ In addition, the double membrane structure is an essential component of the recovery of Gram-negative bacteria, suggesting that tRNA methylation modification is not only involved in the formation but also in the recovery of persisters. It has also been found that an acetyltransferase, TacT, blocks formation of peptide bonds between nascent peptide and tRNA by modifying the primary amine group of amino acids on tRNA molecules, thereby inhibiting protein translation and promoting the formation of persisters;^218^ the accumulated deacylated tRNA trigger bacterial persistence independent of stringent response,^370^ suggesting that tRNA acetylation also plays a role in persister formation. In addition, polynucleotide phosphorylase (PNPase), a component of RNA degradosome (composed of RNase E, helicase RhlB, and enolase together with PNPase), involved in RNA degradation in *E. coli*, has been found to affect the formation of persisters by regulating cAMP/CRP, known to be involved in bacterial metabolism.^38^ Interestingly, we also found that small non-coding RNAs (sRNAs), such as *ryhB*, are also involved in persister formation.^40^ The discovery of these new mechanisms has not only further improved our understanding of the mechanisms of persister formation but also provides new targets for developing anti-persister drugs.

## Compounds or drugs that target and eliminate persister cells

As mentioned above, bacterial persisters not only hinder our ability to effectively treat infections but also act as reservoirs for antibiotic resistant strains. Therefore, killing persisters is crucial for improving treatment outcomes in face of challenges posed by chronic persistent infections, including recurrent infections, bacterial resistance, and biofilm formation. However, the antibiotics commonly used in clinic mainly kill growing bacteria and usually have poor activity against persisters.^371^ As a result, the treatment of chronic persistent infections and biofilm infections becomes exceptionally challenging, resulting in unsatisfactory treatment outcomes.^84,125^ A notable illustration of this challenge is evident in biofilm infections caused by *P. aeruginosa* and *M. abscessus* among individuals with cystic fibrosis. Encouragingly, with the advancement of our understanding of mechanisms and characteristics of persisters, several anti-persister compounds have been discovered^89,372,373^ (Table 2). These compounds can be categorized into three main types: direct killing of persisters, sensitizing persisters to conventional antibiotics, and inhibition of persister formation.

**Table 2:** Compounds/drugs or drug combinations that show anti-persister activity grouped by targeting pathways

Compound/drug or drug combination	Source/origin	Drug target or pathway	Mechanism of action	Target species	Research stage	References
Pyrazinamide (PZA)	Synthetic nicotinamide analog	Metabolism (RpsA, PanD, ClpC1)	Direct killing by inhibiting energy metabolism, trans-translation and protein degradation	*M. tuberculosis*	FDA-approved first-line TB drug	^91^ ^376^
Bedaquiline	Synthetic diarylquinoline	Metabolism (ATP synthase)	Direct killing by inhibiting ATP synthesis	*M. tuberculosis*	FDA-approved second line drug for MDR-TB	^499^
ADEP4	Synthetic acyldepsipeptide	Metabolism (ClpP)	Direct killing by causing a non-specific activation of protease activity	*S. aureus (MRSA)*	In vivo assessment in murine thigh infection model	^177^
Lassomycin/kitamycobactin	Extracts of uncultured soil microbes	Metabolism (ClpC1)	Direct killing by activating ATPase and changing protein degradation profile	*M. tuberculosis*	In vitro assessment	^382^ ^383^
Mavintramycin A	Isolated from the culture broth of *Streptomyces sp*.	Metabolism (23 S ribosomal RNA)	Direct killing by inhibiting protein synthesis	*M. avium complex*	Assessment in silkworm and mouse infection assays	^385^
Carbon metabolite	Mannitol, fumarate glucose, fructose and pyruvate	Metabolism reprogramming	Enhancing drug uptake by generating proton-motive force (PMF)	*E. coli, S. aureus, P. aeruginosa*	Assessment in murine urinary tract infection model or still in vitro	^248^ ^459^ ^460^
Rhamnolipids	Biosurfactant molecules produced by *P. aeruginosa*	Metabolism reprogramming	PMF-independent enhancing drug uptake	*S. aureus including phenotype of SCVs*	In vitro assessment	^461^
N-acetylcysteine	Cysteine	Metabolism	Preventing of persister formation by enhancing respiration	*M. tuberculosis*	Assessment in murine macrophages infection model	^472^
MOPS or osmolytes	Buffer or osmotic protectant	Metabolism	Preventing of persister formation by inhibiting protein aggregation	*E. coli*	In vitro assessment	^473^;
5-aminosalicylic acid	PolyP kinase	Metabolism	Preventing of persister formation by sensitizing bacteria towards oxidative stress by decreasing polyphosphate levels	*E. coli*	In vitro assessment	^474^
Amino acids	serine, L-arginine, glutamine, threonine, glutamine, tryptophan	Metabolism	Sensitizing to antibiotic activity by inhibiting amino acid synthesis, boosting reactive oxygen species (ROS) production and altering membrane pH gradient.	*S. aureus, E. coli, P. aeruginosa*	Assessment in biofilm on a central venous catheter or in vitro	^242^ ^283^ ^464^ ^465^
HT61	Synthetic quinolone-derived compound	Membrane	Disruption of cell membrane	*S. aureus including MRSA, P. aeruginosa*	Phase III trial	^372^
XF-73/ XF-70	A dicationic porphyrin derivative	Membrane	Disruption of cell membrane	*S. aureus including MRSA, C. albicans*	Phase II trial	^394,398–400^
NCK-10	Synthetic aryl-alkyl-lysines	Membrane	Disruption of cell membrane	*S. aureus, E. coli, A. baumannii,* *K. pneumoniae,* *P. aeruginosa,* *C. albicans*	Assessment in murine skin wound infection model	^403^
CD437/CD1530	Synthetic retinoid antibiotics	Membrane	Disruption of cell membrane	*S. aureus*	Assessment in murine chronic thigh infection model	^405^ ^406^
NH125	Synthesized imidazole compound	Membrane	Disruption of cell membrane	*MRSA, MRSE, VRE*	Assessment in *C. elegans* infection model	^398^
Esculentin and its derivatives	Skin membrane-active peptide	Membrane	Disruption of cell membrane	*P. aeruginosa, E. coli O157:H7*	Assessment in sepsis and pulmonary infection models	^413^
PQ401/NTZDpa	Diarylurea derivative	Membrane	Disruption of cell membrane	*MRSA*	Assessment in *C. elegans* and *G. mellonella* infection model	^411^ ^412^
2D-24	Antimicrobial dendrimeric peptide	Membrane	Disruption of cell membrane	*P. aeruginosa*	In vitro assessment	^416^
Piscidin	Fish host-defense peptide	Membrane	Disruption of cell membrane and DNA	*P. aeruginosa*	In vitro assessment	^417^
Trp/Arg-containing peptides	Peptide	Membrane	Disruption of cell membrane	*E. coli*	In vitro assessment	^418^
Art-175	Peptide fused to endolysin	Membrane	Disruption of cell membrane	*A. baumannii,* *P. aeruginosa*	In vitro assessment	^419^ ^420^
QACs	peptide	Membrane	Disruption of cell membrane	*MRSA and E. faecalis*	In vitro assessment	^421^
P14KanS	Kanamycin peptide conjugate	Membrane	Disruption of cell membrane	*S. aureus, S. epidermidis,* *P. aeruginosa,* *A. baumannii*	*A*ssessment in *C. elegans* infection model	^422^
Pentobra	Peptide	Membrane	Disruption of cell membrane	*E. coli, S. aureus*	In vitro assessment	^423^
SPI009	A propanol-amine derivative	Membrane	Disruption of cell membrane	*P. aeruginosa*	In vitro assessment	^426^
PAAG	Polycationic glycopolymer	Membrane	Disruption of cell membrane	*Non-tuberculosis Mycobacterium (NTM)*	In vitro assessment	^426^
AM-0016	A xanthone-based antibacterial	Membrane	Disruption of cell membrane	*M. bovis BCG*	In vitro assessment	^427^
Boromycin	Boron-containing polyether macrolide antibiot	Membrane	Disruption of cell membrane	*M. tuberculosis*	In vitro assessment	^428^
Carvacrol	Essential oil	Membrane	Disruption of cell membrane	*B. burgdorferi*	In vitro assessment	^429^
Lipidated lysine 9	Amino acid	Membrane	Disruption of cell membrane	*E. coli, S. aureus*	In vitro assessment	^430^
Colistin	Repurposed polypeptide antibiotics	Membrane	Disruption of cell membrane	*E. coli*	Assessment in murine urinary tract infection model	^49^
CF-301 (Exebacase)	Recombinant bacteriophage lysin	Cell wall	Direct lysis	*S. aureus*	Completed late-stage clinical trials	^500^
LysH5	Recombinant bacteriophage lysin	Cell wall	Direct lysis	*S. aureus, S. epidermidis*	In vitro assessment	^447^
Engineered bacteriophage	Bacteriophages expressing OmpF	Efflux pump	Modulate sensitivity to antibiotics		In vitro assessment	^448^
Engineered bacteriophage	Bacteriophages expressing the SOS-response repressor lexA3	SOS-response	Reducing persister levels	*E. coli*	Assessment in murine infection model	^448^
M64	Synthetic benzamide-benzimidazole derivatives containing a phenoxy	Cellular communication (MfvR)	Preventing of persister formation by inhibiting MvfR-regulated quorum sensing (QS)	*P. aeruginosa*	Assessment in a murine burned and lung infected model	^467^
Relacin	A potent inhibitor of Rel proteins	Cellular communication (RelA)	Preventing of persister formation by inhibiting RelA -regulated (p)ppGpp signaling pathway	*E. coli, B. subtilis, B. anthracis, Group A streptococci and E. faecalis*	In vitro assessment	^469,470^
BF8	Brominated furanones	Cellular communication (QS)	Reverting persisters by binding signaling molecules	*P. aeruginosa, E. coli*	In vitro assessment	^456^
Mitomycin C	Repurposed anti-cancer drug	DNA cross-link	Leading to spontaneous cross-linking of DNA	*E. coli, S. aureus, P. aeruginosa, A. baumannii, B. burgdorferi*	Assessment in *C. elans* and *G. mellonella* infection model	^449^ ^450^ ^451^
Cisplatin	Repurposed anti-cancer drug	DNA cross-link	Leading to spontaneous cross-linking of DNA	*E. coli, S. aureus, P. aeruginosa*	In vitro assessment	^452^
Anthracyclines	Repurposed anti-cancer drug	-	-	*B. burgdorferi*	In vitro assessment	^453^
Tosufloxacin	Repurposed quinolone antibiotics	DNA gyrase, topoisomerase IV	Damaging DNA replication	*S. aureus*	In vitro assessment	^52^
Clinafloxacin	Repurposed quinolone antibiotics	DNA gyrase, topoisomerase IV	Damaging DNA replication	*S. aureus*	Assessment in murine chronic thigh infection model	^56^
C10	Capric acid	Multiple mechanisms of action	Reverting persisters by enhancing metabolism and damaging membrane	*P. aeruginosa, E. coli*	In vitro assessment	^458^
Metal ions	Silver, iron	Multiple mechanisms of action	Sensitizing to antibiotic activity by disrupting multiple bacterial cellular processes, including disulfide bond formation, metabolism, and iron homeostasis	*E. coli, M. tuberculosis*	Assessment in murine urinary tract infection model	^466^ ^489^
Daptomycin	Repurposed lipopeptide antibiotics	Multiple mechanisms of action	Damaging the cell membrane and wall; Inhibition of protein synthesis	*B. burgdorferi S. aureus*	In vitro assessment	^60^ ^486^
Cis-2-decenoic acid	Fatty acid	Multiple mechanisms of action	Reverting persisters by metabolism, membrane, protein synthesis, communication	*P. aeruginosa, E. coli*	In vitro assessment	^455^
Colistin+Ofloxacin+Nitrofurantoin; Qingdafloxacin + gentamicin + nitrofuran	-	-	Combination of drugs killing both metabolically active bacteria and persisters	*E. coli*	Assessment in a murine urinary tract infection model	^49^ ^501^
Cefoperazone+Doxycycline+ Daptomycin	-	-	Combination of drugs killing both metabolically active bacteria and persisters	*B. burgdorferi*	Assessment in a murine persistent Lyme disease model	^480^
Clinafloxacin+Meropenem+ Daptomycin	-	-	Combination of drugs killing both metabolically active bacteria and persisters	*S. aureus*	Assessment in a murine skin persistent infection model	^56^
Clinafloxacin+Cefuroxime+ Gentamicin	-	-	Combination of drugs killing both metabolically active bacteria and persisters	*P. aeruginosa*	Assessment in a murine cystic fibrosis model	^481^

### Killing of persisters directly

#### Compounds targeting the molecular pathway related to bacterial persistence

As described earlier, multiple processes such as toxin-antitoxin systems, metabolism and metabolic regulatory factors, DNA repair, cell communication, efflux pumps, and epigenetic modifications are involved in the formation and survival of persisters. In these critical processes, targeting the metabolism of trans-translation and protein degradation has emerged as the most successful approach for directly eliminating persisters with anti-persister drugs, including pyrazinamide (PZA), bedaquiline, ADEP4, lassomycin and kitamycobactin.

PZA is a first-line tuberculosis drug that plays a unique role in shortening the duration of tuberculosis chemotherapy. As a well-established prototype anti-persister drug, it has notably decreased the treatment duration for tuberculosis from 9 to 12 months to just 6 months,^91^ highlighting the clinical importance of anti-persister drugs.^17^ It has been found that PZA is a prodrug that is hydrolyzed intracellularly to pyrazinoic acid (POA) by pyrazinamidase (PZase) encoded by *pncA*,^374^ then POA bound to the ribosomal protein S1 (RpsA), a vital protein involved in protein translation and the ribosome-sparing process of *trans*-translation.^29^ PanD, an aspartate decarboxylase, is another target of PZA and that POA binding to PanD could inhibit pantothenate and CoA biosynthesis, which is essential for the central metabolism necessary for energy production and fatty acid metabolism in *M. tuberculosis*.^31,252,375^ In brief, PZA kills *M. tuberculosis* persisters by targeting metabolic processes including trans-translation and protein degradation, as well as energy metabolism.^376^ Bedaquiline, also known as TMC207, is a new antimycobacterial agent recently approved by the FDA for treating pulmonary MDR-TB by inhibiting ATP synthase.^377^ Research has shown that when combined with other agents such as pretomanid and moxifloxacin, bedaquiline effectively eliminates *M. tuberculosis* in non-replicating persister state.^378,379^ This indicates that bedaquiline may serve as an anti-persister drug against *M. tuberculosis*, similar to PZA.

ADEP4 is a compound against *S. aureus* persisters targeting ClpP ATPase, which was isolated from the fermentation broth of *Streptococcus hawaiiensis* NRRL 15010 ^177,195,380,381^. The combination of ADEP4 with rifampicin has been demonstrated to achieve complete eradication of *S. aureus* biofilm persisters in both in vitro and in vivo studies.^177^ Though, this was not confirmed in a recent study using a more persistent infection model.^56^ Nevertheless, it is worth highlighting that the effectiveness of an antimicrobial drug does not solely depend on targeting essential components for bacterial viability. Antimicrobial drugs that target previously unexplored pathways, such as activating the ClpP protease, have shown no cross-resistance to any antibiotic classes currently available or in development. This unique characteristic makes them an ideal candidate for combination therapy in the treatment of infections caused by drug-resistant or drug-tolerant bacteria. Lassomycin^382^ and its analog kitamycobactin,^383^ which were screened from extracts of uncultured soil microbes, have also shown activity against *M. tuberculosis* persisters. Their targets have been identified as the Clp ATPase complex as well. Specifically, lassomycin binds to a highly acidic region of the ClpC1 ATPase, leading to ATPase activation and changes in protein degradation profile.^384^ These findings suggest that exploring anti-persister drugs based on the molecular mechanisms of bacterial persistence, especially metabolism, holds promise for future research.

In summary, in addition to the above compounds, there are still compounds targeting other molecular mechanisms of bacterial persistence. For instance, mavintramycin A was found to be active against persistent infection of *M. avium* complex (MAC) by binding to 23S ribosomal RNA and inhibiting protein synthesis.^385^ However, although many molecular mechanisms of bacterial persistence have been identified and validated over time, these persister drugs mainly target metabolism. Therefore, in future studies, it is crucial to explore the development of more anti-persister drugs targeting other essential molecular mechanisms involved in the formation and survival of persisters.

#### Membrane active small molecules

Another prominent target for directly attacking persisters is the bacterial membrane.^386^ HT61 is a novel quinolone-derived compound against non-multiplying persisters, including methicillin sensitive and resistant *S. aureus*.^387,388^ HT61 has also been proposed as an adjunct to other antimicrobials to extend their usefulness. For example, it has been shown to enhance the effect of tobramycin against *P. aeruginosa* both in vitro and in vivo.^389^ Additionally, HT61 has been found to enhance the activity of neomycin, gentamicin, mupirocin, and chlorhexidine against both methicillin-susceptible *S. aureus* (MSSA) and methicillin-resistant *S. aureus* (MRSA) in vitro and in vivo.^390^ It is worth mentioning that HT61 nasal gel has also shown promising results in Phase II clinical trials (EudraCT number, 2009-017398-39) and Phase III trials (EudraCT number, 2010-021193-11) by enhancing the effectiveness of conventional antibiotics in eradicating persistent nasal colonization of *S. aureus*.^372^ The mechanism of action of HT61 involves non-specific targeting of anionic lipids that are abundant in bacterial membranes.^391,392^ By interacting with these lipids, HT61 disrupts the integrity of the bacterial cell membrane, leading to cell lysis and release of ATP from the bacterial cells.

XF-73 is another membrane-active agent, porphyrin compound, with rapid bactericidal activity, which is active against both slow-growing and non-dividing cultures of *S. aureus* including biofilms.^393–396^ In a randomized, open-label, Phase I clinical trial, nasal formulations of XF-73 demonstrated good safety and local tolerability.^397^ In a Phase II clinical trial, the intranasal administration of XF-73 24 h prior to surgery significantly reduced *S. aureus* nasal carriage in patients undergoing cardiac surgery.^398^ These findings suggest the potential of XF-73 as an effective intervention to reduce *S. aureus* colonization and associated infections in surgical settings. Further research has revealed that XF-73 also exhibits antimicrobial effects against *Candida albicans* (*C. albicans*) and its biofilms.^399,400^ This expands the potential applications of XF-73 beyond its activity against *S. aureus*, highlighting its broad-spectrum antimicrobial properties. Continued investigation is necessary to fully understand the extent of XF-73’s antimicrobial activity and its potential clinical applications. XF-70, a compound structurally similar to XF-73, has also demonstrated the ability to combat slow-growing and non-dividing cultures of *S. aureus* including biofilms.^394^ Furthermore, XF-70 has exhibited additional activity against small-colony variant *hemB* mutants of MSSA and MRSA.^401^

NCK10 is an aryl-alkyl-lysine compound with a decyl chain appendage that has been shown to effectively lyse persister cells of *E. coli* and biofilms of *A. baumannii* (MTCC 1425), *E. coli* (MTCC 443), *K. pneumoniae* (ATCC 700603) and *P. aeruginosa* (MTCC 424) in murine model of burn infection.^402^ Furthermore, NCK10 has exhibited activities against planktonic cells, persister cells, biofilms of MRSA, and has shown the potential to protect mice from skin infections.^403^ Additionally, NCK10 has displayed broad-spectrum antibacterial activity, demonstrating potency against both immature and mature biofilms of *C. albicans*.^404^ This compound achieves its antimicrobial effects by depolarizing and permeabilizing the bacterial outer membrane, leading to cell lysis. These findings suggest that NCK10 holds potential as a broad-spectrum antimicrobial agent against persistent bacterial infections and biofilms. Further research is needed to fully understand the mechanism of action and potential clinical applications of NCK10.

Another class of membrane-active agent against persisters is synthetic retinoid derivatives. Two notable representatives of this class are CD437 and CD1530, which were identified through a screening of approximately 82,000 compounds using a *C. elegans*-MRSA infection model.^405^ Both CD437 and CD1530 have demonstrated the ability to eliminate persisters formed in MRSA biofilms by disrupting the bacterial membrane bilayer and inducing membrane permeabilization.^406^ Furthermore, an analogue of CD437 has shown antimicrobial activity against MRSA persisters while exhibiting improved cytotoxicity compared to CD437.^406^ In a MRSA mouse deep-seated thigh infection model, CD437 or its analogue, either alone or in combination with gentamicin, exhibited promising efficacy.^406^ These findings highlight the potential of synthetic retinoid compounds as effective agents against persisters and their potential for combination therapy with conventional antibiotics.

NH125 (1-hexadecyl-2-methyl-3-[phenylmethyl]-1H-imidazolium iodide), a bacterial histidine kinase inhibitor, was identified using SYTOX Green screening assay. It was found to possess strong bactericidal properties against MRSA persisters by inducing cell membrane permeabilization.^407,408^ Furthermore, NH125 has demonstrated either no toxicity or low toxicity to *C. elegans*.^407^ In addition, several analogues of NH125 have been identified to possess the ability to kill MRSA, methicillin-resistant *Staphylococcus epidermidis* (MRSE), and vancomycin-resistant *Enterococcus faecium* (VRE) persisters and biofilms and eradicate fungal biofilms.^409,410^ These findings suggest that NH125 and its analogues hold potential as promising drug candidates for combating persistent bacterial and fungal infections. In addition to NH125, several other membrane-active small molecules, such as PQ401^411^ and NTZDpa,^412^ have shown promising activity against MRSA persisters in in vitro studies or in models like *C. elegans* and *Galleria mellonella*. These compounds have demonstrated potential as effective agents against persistent MRSA infections. Further research is needed to evaluate their efficacy, safety, and potential for clinical use.

From the above information, it is evident that the currently discovered membrane-active small molecules have primarily been studied for their activity against *S. aureus*, including drug-resistant strains and biofilms. However, among these compounds, NCK10 stands out with its relative broad-spectrum antibacterial activity. NCK10 has demonstrated efficacy not only against Gram-positive bacteria like MRSA but also against Gram-negative bacteria such as *A. baumannii*, *E. coli*, *K. pneumoniae* and *P. aeruginosa*. Except for NCK10, esculentin (1–21), an amphibian skin membrane-active peptide, has also shown activity against both planktonic and biofilm cells of *P. aeruginosa* and prolong survival of animals in models of sepsis and pulmonary infection.^413^ Derivatives of esculentin peptides have exhibited efficacy against infections caused by *E. coli* strains such as O157:H7.^414,415^ Furthermore, numerous other peptides have been identified as anti-persister drugs. These include 2D-24^416^ and piscidin^417^ targeting *P. aeruginosa*, Trp/Arg-containing peptides effective against *E. coli*,^418^ Art-175 (fusion of the sheep myeloid 29-amino acid peptide and the KZ144 endolysin) effective against *A. baumannii*^419^ and *P. aeruginosa*,^420^ quaternary ammonium cations (QACs) effective against MRSA and *E. faecalis*,^421^ P14KanS effective against *S. aureus* and *S. epidermidis*, as well as *P. aeruginosa* and *A. baumannii*,^422^ and pentobra effective against *E. coli* and *S. aureus*.^423^ These broad-spectrum activities highlight the potential of peptides as versatile antimicrobial agents capable of targeting a range of bacterial pathogens. Additionally, a propanol-amine derivative (1-((2,4-dichlorophenethyl) amino)-3-phenoxypropan-2-ol), known as SPI009, also showed broad-spectrum activities against persister cells of various Gram-positive and Gram-negative pathogens by inducing significant membrane permeabilization.^424,425^

Polycationic glycopolymer, such as poly (acetyl, arginyl) glucosamine (PAAG), represent another type of membrane-active compound against mycobacteria. Studies have shown that PAAG treatment can effectively eradicate antibiotic-induced persister cells in planktonic cultures of non-tuberculosis mycobacteria (NTM).^426^ Additionally, PAAG has demonstrated the ability to disperse NTM biofilms.^426^ These findings suggest that PAAG holds promise as a potential therapeutic agent for combating persistent NTM infections. However, its efficacy has only been demonstrated in in vitro experiments. Furthermore, there are many other membrane active molecules exhibiting effective anti-persister activity are currently under evaluation in vitro. For example, 3,6-Di[4-(diethylamino)-butoxy]-1-hydroxy-7-methoxy-2,8-bis(3-methylbut-2-enyl)-9H-xanthen-9-one (AM-0016)^427^ and boromycin^428^ kills mycobacterial persisters; carvacrol (2-methyl-5-(1-methylethyl) fenol) showed activity against *B. burgdorferi* persisters;^429^ lipidated lysine 9, was capable of lysing persister cells of *E. coli* and *S. aureus*.^430^ Further research is needed to evaluate the activity of these agents.

Overall, disrupting the bacterial membrane bilayer has emerged as a promising strategy for killing persisters. While many membrane-active compounds have demonstrated good activity against persisters, and some even show promising clinical application prospects, there are several obstacles that need to be considered during the discovery and assessment of these agents. For instance, potential candidates have been found to effectively kill pathogens, but they may also disrupt mammalian membranes and have potential toxicity.^431,432^ Therefore, structural transformation and optimization are necessary to develop species-specific molecules. NH125 serves as an example of this, as it exhibits low cytotoxicity due to careful design and optimization.

#### Phage-derived therapy

Phage therapy is an ancient anti-infective tool that predates even penicillin.^433^ However, the challenges of phage therapy include insufficient pharmacokinetic and pharmacodynamic studies, the need for further evaluation of clinical efficacy, and the management of foreign proteins, nucleic acids, and virulence factors introduced by phages, all of which pose significant obstacles to its widespread implementation.^433,434^ In recent years, phage therapy has been sporadically approved for compassionate use, which refers to emergency use when no other approved therapy is available.^435,436^ Furthermore, phage therapy has shown promising therapeutic outcomes in the majority of cases.^437–439^ Therefore, studies on phage therapy for anti-infective treatments, especially drug-resistant infections, have been increasing.^439–442^

Research on phage therapy for the treatment of persistent infections has also made some progress. The most advanced development is the recombinant bacteriophage lysin CF-301, known as PlySs2 or exebacase, which is the first agent in this category to complete late-stage clinical trials (phase 3, NCT04160468) for the treatment of *S. aureus* bacteremia, including right-sided endocarditis.^443^ CF-301 was first cloned by the team of Vincent Fischetti, with the gene derived from a *Streptococcus suis* (*S. equi*) phage sequence. It was found that the chromatographically purified CF-301 exhibited broad lytic activity against various bacteria, including MRSA, vancomycin-intermediate *S. aureus* (VISA), *Streptococcus suis*, Listeria, *Staphylococcus simulans*, *Staphylococcus epidermidis*, *S. equi*, *Streptococcus agalactiae*, *Streptococcus pyogenes*, *Streptococcus sanguinis*, group G streptococci (GGS), group E streptococci (GES), and *Streptococcus pneumoniae*.^444^ Subsequently, the antibacterial activity of CF-301 against *S. aureus* persister cells in exponential-phase and stationary-phase populations and biofilm was further demonstrated.^445,446^ Additionally, the endolysin LysH5 derived from the phage vB_SauS-phiIPLA88 induces cell lysis in both actively growing and non-growing *S. aureus* and *S. epidermidis*, enabling the elimination of persister cells.^447^ Both of these therapies, derived from phages, act through the mechanism of cell wall hydrolases. Moreover, engineered bacteriophages expressing the outer membrane protein OmpF enhanced the eradication of persister cells by fluoroquinolones compared to monotherapy.^448^ Another type of bacteriophage, expressing the SOS-response repressor LexA3, also successfully reduced *E. coli* persister levels.^448^

#### Repurposed existing anti-cancer drugs or antibiotics

Moreover, existing anti-cancer drugs or antibiotics were re-purposed to kill bacterial persisters, which have the advantage of saving the cost and time of new drug research and development. For example, mitomycin C (MMC), an FDA-approved anti-cancer drug, has been found to effectively eradicate *E. coli*, *S. aureus* and *P. aeruginosa* persisters both in vitro and in vivo,^449^ as well as *A. baumannii*^450^ and *B. burgdorferi*^451^ persisters. Likewise, another FDA-approved anti-cancer drug, cisplatin, has demonstrated the ability to eradicate persister cells of *E. coli*, *S. aureus*, and *P. aeruginosa* as well.^452^ The antimicrobial mechanisms of these two drugs involve causing growth-independent crosslinking of DNA, thereby effectively eliminating persister cells.^449^ In addition, anti-cancer drug anthracyclines were also found to be capable of killing *B. burgdorfer*i persisters,^453^ although the exact mechanism of action remains to be determined they may act by DNA damage. Nevertheless, it should be pointed out that the high toxicity of anti-cancer drugs may affect their applications in clinic as anti-persister therapies. In addition, there are also known antibiotics that have been repurposed for persister killing, such as tosufloxacin,^52^ colistin,^49^ clinafloxacin,^56^ daptomycin^60^ etc., which have the advantage of not requiring additional clinical safety evaluations.

### Sensitizing persisters to conventional antibiotics

Due to the inactive metabolic state of persisters, conventional antibiotics are rarely effective against persisters. However, researchers have discovered that strategies such as reverting or awakening persisters, enhancing antibiotic uptake or increasing bactericidal action of antibiotics can render them susceptible to conventional antibiotics.

#### Reverting or awakening persisters

Reversing or awakening persisters to their normal bacterial states, which respond to antibiotic treatment, is an ideal approach for studying anti-persister drugs. While resuscitation factors have been identified for bacterial resuscitation, the practical implementation of reverting or awakening persisters remains limited. It has been observed that the addition of spent medium containing protein and peptide factors resuscitated *S. aureus* persister cells, enhancing their susceptibility to antibiotic-induced eradication.^454^

Recently, the fatty acid signaling molecule cis-2-decenoic acid has been shown to induce persisters of both *P. aeruginosa* and *E. coli* to transition into ciprofloxacin-susceptible states, but the mechanism involved is still unclear.^455^ Another compound, BF8, was also found to revert antibiotic tolerance of *E. coli* and *P. aeruginosa* persister cells, although the underlying mechanism remains unknown.^456,457^ In addition, 3-[4-(4-methoxyphenyl)piperazin-1-yl]piperidin-4-ylbiphenyl-4-carboxylate (C10), identified from a chemical library screen, demonstrated the ability to reverse the persister phenotype of both *P. aeruginosa* and *E. coli* to antibiotic-sensitive cells by enhancing their metabolism.^458^ This action rendered these tolerant cells susceptible to various classes of antibiotics.

#### Enhancing drug uptake

Carbon metabolites were first discovered to promote the uptake of aminoglycoside antibiotics in persisters. Allison et al. demonstrated that carbon metabolites like glucose, mannitol, fructose and pyruvate can increase the generation of proton-motive force (PMF), thereby promoting the uptake of aminoglycosides in persisters.^248^ This mechanism facilitates the killing of persisters by aminoglycosides in both *E. coli* and *S. aureus*.^248^ This work establishes a metabolism reprogramming strategy for eradicating bacterial persisters and emphasizes the significance of the metabolic environment in antibiotic treatment. Furthermore, other studies have also found that mannitol (10–40 mM) can increase the sensitivity of *P. aeruginosa* persister cells to tobramycin by up to 1000-fold.^459^ Similarly, fumarate has been shown to have a similar effect.^460^ Rhamnolipids, which are biosurfactant molecules produced by *P. aeruginosa*, have been found to enhance the uptake and activity of aminoglycosides against *S. aureus* persisters including phenotype of SCVs.^461^ It is worth mentioning that rhamnolipids promoting the uptake of aminoglycosides in persisters is PMF-independent, unlike carbon metabolites.

Moreover, specific environmental factors have also been found to sensitize persistent bacteria to antibiotics by increasing drug uptake. For instance, a two-minute treatment under hypoionic shock conditions (e.g., in pure water) significantly enhances the bactericidal effects of aminoglycosides against both spontaneous and triggered *E. coli* persisters.^462^ This enhancement may be attributed to the involvement of mechanosensitive channels (MS). Additionally, freezing has been observed to dramatically increase the bacterial uptake of aminoglycosides independent of PMF.^463^ This effect may be attributed to freezing-induced cell membrane damage, such as the activation of the mechanosensitive ion channel MscL. These findings provide valuable insights into the development of new strategies against bacterial persisters by combining existing antibiotics with MS or MscL agonists. Such combination approaches have the potential to enhance the efficacy of antibiotics and overcome the challenges posed by persistent bacterial infections.

#### Potentiating antibiotic activity

Amino acids have also been found to enhance antibiotic-mediated killing through several pathways, including inhibiting amino acid synthesis, boosting endogenous reactive oxygen species (ROS) production, and altering membrane pH gradient. For instance, supplying amino acids (such as serine, threonine, glutamine, and tryptophan) to wild-type *E. coli* sensitizes stationary-phase cells to gentamicin, potentially by inhibiting amino acid synthesis.^242^ Additionally, the addition of serine can enhance the effectiveness of ofloxacin or moxifloxacin against *E. coli* by increasing NADH production.^464^ This strategy works by elevating the NAD (+)/NADH ratio, disrupting Fe-S clusters, and boosting the generation of endogenous ROS. Furthermore, L-arginine enhanced gentamicin activity against *S. aureus*, *E. coli* and *P. aeruginosa* persisters by modifying the membrane pH gradient.^465^ Moreover, glutamine has been found to promote antibiotic uptake, leading to killing of multidrug-resistant uropathogenic bacteria.^283^ Our previous studies have demonstrated the significant role of glutamate in the formation and survival of bacterial persistence.^286^ Therefore, facilitation of these amino acids may hold promise for future development of effective approaches to manage chronic bacterial persistent infections.

Besides amino acids, metal ions could also potentiate antibiotic activity against persisters by enhancing ROS production. It has been found that combinations of silver and antibiotics eradicate Gram-negative bacterial persisters both in vitro and in vivo.^466^ Additionally, iron has been shown to enhance the activity of the persister drug PZA against *M. tuberculosis*. With recent progress in nanotechnology and surface chemistry, it is conceivable to create silver nanoparticles with antibiotic-functionalized surfaces embedded in medical materials that regulates the release of active antimicrobial agents at the infection site.

### Inhibiting persister formation

Unlike strategies that specifically aim to eliminate persisters to cure persistent infections, an alternative strategy for addressing persistent infections involves preventing the formation of persisters in the first place. By interfering with the mechanisms involved in persister formation, it may be possible to prevent the development of persistent infections. Specifically, targeting quorum sensing (QS) and (p)ppGpp, as mentioned earlier, can be effective as they play crucial regulatory roles in persister formation.

Researches have demonstrated that inhibiting the transcriptional regulator MfvR in QS system with a synthetic benzamide-benzimidazole derivative (referred to as M64) is effective against persistent *P. aeruginosa* infections both in vitro and in vivo.^467,468^ This compound represents the first identified to decrease the formation of antibiotic-tolerant persister cells. In addition, another compound, relacin, which targets the (p)ppGpp signaling pathway, has been shown to reduce persister levels in *E. coli*, *B. subtilis*, *B. anthracis*, Group A streptococci and *E. faecalis*.^469,470^ Relacin works by preventing the accumulation of the alarmone (p)ppGpp through the inhibition of RelA, thereby disrupting the bacterial stringent response.^469^ The stringent response is a key pathway involved in bacterial persistence which is mediated by the alarmone (p)ppGpp. In *M. tuberculosis*, it has been found that targeting various components of the stringent response could lead to altered antibiotic susceptibility and potentially shorten tuberculosis treatment.^471^ These suggest that intervening in QS and (p)ppGpp pathways can potentially disrupt the formation of bacterial persisters, thereby assisting in the treatment of infectious diseases. Although these compounds have not yet entered clinical application, these research findings bring hope for the treatment of persistent bacterial infections.

In addition to QS and (p)ppGpp, other pathways could also be used in controlling persister formation. For example, enhancing respiration and thus metabolism using N-acetylcysteine reduced the formation of *M. tuberculosis* persisters;^472^ inhibiting the accumulation of protein aggregates by MOPS (3-(N-morpholino) propanesulfonic acid) or osmolytes (such as betaine, trehalose, glucose, and glycerol) reduced the frequency of persister formation;^473^ increasing bacterial sensitivity to oxidative stress by 5-aminosalicylic acid (also known as mesalamine) is also capable of reducing persister formation;^474^ or interfering with the bacterial response to oxidative stress by using bacteriophages expressing the SOS-response repressor LexA3 increased persister killing as well.^448^ Therefore, the growing understanding of the mechanisms underlying bacterial persistence has resulted in the discovery of additional strategies that disrupt processes involved in persister formation, consequently reducing the number of persisters.

## Proposal of persister drug combination approach for more effective treatment of persistent infections

The earliest treatment strategy for persistent infections can be traced back to the 1940s, coinciding with the first description of the phenomenon of bacterial persisters.^62^ Joseph Bigger suggested that intermittent or pulse antibiotic use would enable surviving persister cells to resuscitate during non-treatment periods, followed by their rapid elimination during subsequent treatment. However, this approach is difficult to use due to unpredictable resuscitation of the persisters in vivo and has not been used clinically. In the following clinical treatment of challenging persistent infections like tuberculosis^17,378^ and acquired immune deficiency syndrome (AIDS),^475,476^ combination therapies with multiple drugs are employed, which may also be the most effective approach for treating other persistent infections. This proposal aligns with the findings and recommendations of other scientists,^88,477–479^ and its validity has been further supported by recent animal studies. For example, chronic persistent infections caused by *B. burgdorferi*, *S. aureus*, *P. aeruginosa* and *E. coli* can be effectively eradicated using this approach by applying different drug combinations (cefoperazone + doxycycline + daptomycin;^480^ clinafloxacin + meropenem + daptomycin;^56^ clinafloxacin + cefuroxime + gentamicin;^481^ colistin + ofloxacin + nitrofurantoin^49^) in respective animal models. Further clinical evaluations are needed to see if these antibiotic combinations can eradicate the corresponding persistent infections in patients. However, it is important to note that different antibiotics have distinct mechanisms of action and can sometimes exhibit antagonistic effects on each other^482^ or promote the emergence of tolerance.^483^ Therefore, it is necessary to continue to search for rational drug combinations that can effectively kill persisters and cure persistent infections.

**Fig. 7:**
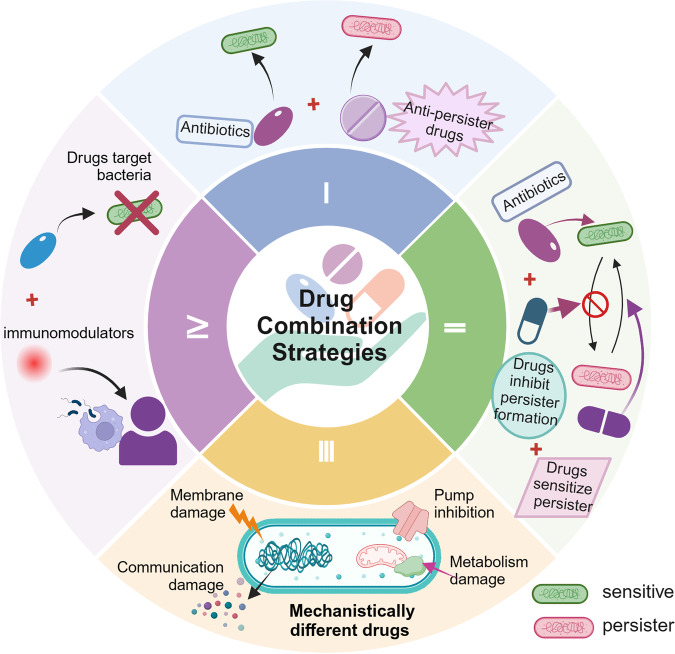
Proposed drug combination strategies for treating persistent infections. (1) Combination of drugs killing both metabolically active bacteria and persister cells per Yin-Yang model 24. (2) Compounds sensitizing persisters or inhibiting persister formation combined with traditional antibiotics. (3) Utilizing mechanistically diverse drugs to potentially eliminate the persister population. (4) Combining antibacterial agents and immunomodulators to modulate host immunity. Created with BioRender.com

Firstly, combination of drugs killing both metabolically active bacteria and persister cells (Fig. 7). In theory, due to the complex heterogeneous bacterial population consisting of growing and non-growing persister cells,^24,484,485^ a drug combination approach targeting these two bacterial populations would be important to cure persistent infections, according to the Yin-Yang model.^24,477^ The clinical experience with the persister drug PZA to kill persisters and shorten the treatment of tuberculosis without relapse, indicates that using a combination of drugs that not only kill growing bacteria but more importantly kill the persisters would more effectively cure persistent infections.^24^ Additionally, the anti-persister drug daptomycin^486^ and colistin,^49,487^ when used in combination with other conventional antibiotics, has demonstrated effective eradication of the aforementioned persistent infections,^49,56,480^ further underscoring the success of the persister drug combination approach.^24^ Moreover, in combination with conventional antibiotic rifampin, ADEP4 was effective against a deep-seated murine *S. aureu*s biofilm infection,^177^ although there is still debate over whether it could completely clear persistent infections.^481^

Secondly, combination of compounds that sensitize persisters or inhibit persister formation with traditional antibiotics^488^ (Fig. 7). This approach takes into account the complex interactions between antibiotics and persister cells, aiming to enhance the effectiveness of treatment by traditional antibiotics. Currently, there are several compounds that sensitize persister cells, such as cis-2-decenoic acid,^455^ carbon metabolites,^248,459,460^ amino acids,^242,283,464,465^ metal ions,^466,489^ and others. However, their effectiveness in clearing persistent infections still needs further validation in vivo.

Additionally, the combination of mechanistically different drugs also has the potential to eradicate the persister population (Fig. 7). When bacteria are exposed to stressful environments, they employ various strategies to ensure their survival, such as forming metabolically slow or dormant persister cells. Through the elucidation of the bacterial persister mechanisms mentioned above, it becomes evident that persister formation is a result of multiple factors working in concert, rather than being caused by a single factor. Therefore, it is speculated that the use of anti-persister drugs targeting a single target may not fully control persister cells.

Finally, combination of antibacterial agents and immunomodulators targeting the host immunity (Fig. 7). The formation of persisters is influenced not only by environmental stresses such as antibiotic, temperature, pH, and nutrients in vitro, but also influenced by host factors in vivo.^490,491^ For example, it has been found that the internalization of *Salmonella typhimurium* by macrophages triggers the formation of non-replicating persister cells.^491,492^ The observations with *S. aureus*^493,494^ and uropathogenic *E. coli* persisters^495,496^ in host cells further illustrate the impact of host factors. These findings suggest that modulating host immunity could be effective in combating persistent infections. This idea was confirmed by the finding that inactivated vaccine, such as the Salmonella cells killed by peracetic acid, reduces the formation of reservoirs of persisters after oral infection with *S. typhimurium*.^172^ Moreover, DNA vaccine^497^ and cytokine granulocyte macrophage colony-stimulating factor (GM-CSF)^498^ also showed activities against persistent infections. Under these conditions, the use of immunotherapy alongside traditional chemotherapeutic antibacterial drugs could potentially lead to a quicker or more definitive resolution of persistent infections in humans.

## Conclusion and future perspective

In this review, we comprehensively summarized the historical background of bacterial persisters, including its discovery in vitro, validation through clinical isolation and in animal models. We detailed the complex characteristics of persister bacteria and argue their relationship with tolerant and resistant bacteria. Additionally, we systematically addressed the interplay between various bacterial biological processes and the formation of persister cells. Moreover, we consolidate the diverse anti-persister compounds and therapies documented in the literature to date. We conclude that the widespread recognition of the clinical significance of persistent infections has driven research on persister cells, resulting in a deeper understanding of their mechanisms and potential treatment strategies. Nevertheless, there remain numerous unanswered questions regarding the molecular mechanism and treatment strategy of persisters that necessitate further exploration and discussion.

For example, while in vitro screening of genes associated with persisters is primarily carried out under the action of antibiotics, there are many other factors (such as oxygen, pH, nutrient starvation and other environmental factors such as those encountered in the host) that play a role in the formation of persisters but have not been well studied. Moreover, the relative importance and interaction of different persistence genes and pathways under different conditions are very complex, and many details in regulating the formation and survival of persisters are still unclear^189,190^ and need further study. The molecular basis for the heterogeneous nature of persisters from shallow to deep persistence to VBNC in the continuing spectrum of persisters also still remains to be investigated.^24^ Additionally, different host-specific niches can promote persistence in the context of infection, but our understanding of this mechanism is far from complete. Further elucidation of these unclear mechanisms of bacterial persistence should help to provide new targets for development of new anti-persister drugs. Upon reviewing the mechanisms of persistence, it becomes evident that persister bacteria do not arise from a single factor or gene regulation. Therefore, it is imperative to identify key synthetic lethal gene combinations that impact persister formation or survival as potential drug targets for future intervention studies.

Regarding drug development for persisters, there still remain significant challenges and a long road ahead. For instance, although researchers have previously developed compounds or drug candidates that directly target and kill persisters, such as ADEP4, providing hope for the treatment of persistent bacteria, the experimental treatments using ADEP4 + rifampin have failed to effectively clear persistent infections.^481^ Perhaps the most effective strategy is to employ persister drug combinations per the Ying-Yang model, such as drugs targeting growing cells + drugs targeting persister cells, or drugs sensitizing persister cells or preventing persister formation + traditional antibiotics. Furthermore, it has been suggested that during long-term persistent infections, bacterial persisters emerge and adapt within a complex host environment,^490^ indicating potential involvement of the immune system in this process. Therefore, modulating the host immune system and host environment could hold importance for more efficacious treatment of chronic persistent infections. For instance, therapeutic vaccines could be developed based on antigenic key proteins of persisters. Additionally, Traditional Chinese Medicines or extracts with immunomodulatory effects could be employed to regulate the host immune microenvironment, enhancing the immune response against persistent bacteria.

From the mechanism in Table 1 to the drugs in Table 2, we found that the drug mechanisms of killing persisters mainly focus on altering metabolism, disrupting membranes, and DNA cross-linking, while the drug mechanisms of sensitizing persisters to conventional antibiotics or inhibiting persister formation primarily concentrate on stress response and cell communication. There are still many persister mechanisms that have not been utilized for drug development, indicating significant potential and opportunities for the development of anti-persister drugs. It is encouraging that the persister drug combination approach, developed per the Yin-Yang model based on the persister drug PZA, has produced promising results to more effectively kill persisters and biofilm bacteria not only in vitro but also cure the persistent infections in animal models in vivo.^56,480,481^ Further studies are warranted to validate this approach for more effective treatment of persistent infections in clinical studies.
